# Evolution of rubisco complex small subunit transit peptides from algae to plants

**DOI:** 10.1038/s41598-017-09473-x

**Published:** 2017-08-24

**Authors:** Md. Abdur Razzak, Dong Wook Lee, Yun-Joo Yoo, Inhwan Hwang

**Affiliations:** Division of Integrative Biosciences and Biotechnology, Pohang University of Science and Technology, Pohang, 37673 Korea

## Abstract

Chloroplasts evolved from a free-living cyanobacterium acquired by the ancestor of all photosynthetic eukaryotes, including algae and plants, through a single endosymbiotic event. During endosymbiotic conversion, the majority of genes in the endosymbiont were transferred to the host nucleus and many of the proteins encoded by these genes must therefore be transported into the chloroplast after translation in the cytosol. Chloroplast-targeted proteins contain a targeting signal, named the transit peptide (TP), at the N-terminus. However, the evolution of TPs is not well understood. In this study, TPs from RbcS (rubisco small subunit) were compared between lower and higher eukaryotes. *Chlamydomonas reinhardtii* RbcS (CrRbcS) TP was non-functional in Arabidopsis. However, inclusion of a critical sequence motif, FP-RK, from *Arabidopsis thaliana* RbcS (AtRbcS) TP allowed CrRbcS TP to deliver proteins into plant chloroplasts. The position of the FP-RK motif in CrRbcS TP was critical for function. The QMMVW sequence motif in CrRbcS TP was crucial for its transport activity in plants. CrRbcS TPs containing additional plant motifs remained functional in *C. reinhardtii*. These results suggest that TPs evolved by acquiring additional sequence motifs to support protein targeting to chloroplasts during evolution of land plants from algae.

## Introduction

Chloroplasts are found in a diverse range of eukaryotes, from single-celled organisms such as algae to multicellular higher plants. However, all chloroplasts are derived from an endosymbiotic organelle that evolved from a free-living cyanobacterium after a single endosymbiotic event with a cyanobacterium approximately 1 billion years ago^1^. In endosymbiotic conversion, one earliest evolutionary event to occur after endosymbiosis was transfer of genes from the endosymbiont to the nucleus of the host. Subsequently, proteins encoded by the transferred genes were provided to the endosymbiont after transcription and translation in the host cytosol. Thus, establishing protein import mechanisms by which the endosymbiont undergoing organellogenesis could obtain proteins from the host can be considered the most crucial event that gave rise to chloroplast evolution. The successful import of proteins from the host cytosol to the endosymbiont might have made possible for further transfer of genes from the endosymbiont to the host. In addition, the import of the encoded proteins from the host into the endosymbiont over evolutionary time might have led to the conversion of a free-living bacterium to an organelle. Chloroplasts in present-day higher plants contain less than 100 genes in their genomes^2^ and import approximately 3000 proteins that are encoded by nuclear genes and translated in the cytosol^3–5^.

Host cell transcription and translation mechanisms were exploited to produce proteins for the nascent chloroplast. However, sorting of chloroplast proteins in the cytosol and translocation through the outer and inner envelope membranes originated from double membrane layer in cyanobacteria must have required the establishment of new mechanisms during endosymbiotic conversion of the cyanobacterium. Currently, an N-terminal transit peptide (TP), defined by the sequence composed of N-terminal region (cTP) cleaved off by SPP (stromal processing peptidase) and some part of mature region, is both necessary and sufficient for targeting proteins to the chloroplast^6–11^. However, it is not fully understood how chloroplast proteins acquired TPs, and whether and how TPs were modified over evolutionary time. TPs are recognized by molecular machinery localized at chloroplast envelope membranes, and one possible scenario is that TPs coevolved with the molecular components involved in the import process.

Photosynthetic eukaryotes share common translocons for import of chloroplast proteins. Small sequence motifs found throughout the TP play key roles in import into chloroplasts, probably by acting as binding or recognition motifs for various translocon components in Toc/Tic complexes^6–9, 12^. However, TP sequences are not highly conserved in length or amino acid sequence^13^. The factors underlying the differences among different types of chloroplast proteins are not fully understood.

RbcS, which is involved in CO_2_ fixation and is one of the most important chloroplast proteins, is encoded by nuclear gene(s). In addition, RbcS TP has been used as a model to study how TPs can support specific protein import into chloroplasts^6–12, 14^. In this study, RbcS TPs from Chlamydomonas and Arabidopsis were used to examine TP development during the evolution of land plants from algae. TP capacity to deliver proteins into chloroplasts was compared and used to model TP evolutionary development. Wild-type TP from *Chlamydomonas reinhardtii* RbcS (CrRbcS) was unable to deliver proteins to chloroplasts in Arabidopsis. However, a small critical sequence motif in the TP of *Arabidopsis thaliana* RbcS (AtRbcS) was able to complement the defect and allow CrRbcS TP to function in Arabidopsis. These results strongly suggest that plant TPs evolved from corresponding algal TPs by acquisition of new sequence motifs during evolution.

## Results

### CrRbcS TP is not functional in Arabidopsis

To gain insights into TP evolution, initially, sequence similarities were examined between 83 plant and 33 algal RbcS TPs (Table S1). Alignment of TP sequences from RbcS in algae and higher land plants revealed that RbcS TPs showed a high degree of similarity among higher land plant TPs and also among algal TPs but not between plant and algal TPs (Fig. S1). Moreover, phylogenetic analysis indicates that all algal RbcS TPs were grouped into a clade to which RbcS TPs from land plants do not belong (Fig. S2). The proposed Toc34-interacting motifs FGLK and FP-RK (highlighted in yellow) were not clearly defined in algal RbcS TPs^8, 12^. Moreover, the fifth 10-amino-acid (T5) segment (DITSITSNGG)^6^ in plant TPs (highlighted in green) was almost absent in algal TPs, and algal RbcS TPs were thus shorter in length than plant RbcS TPs (Fig. S1).

**Figure 1:**
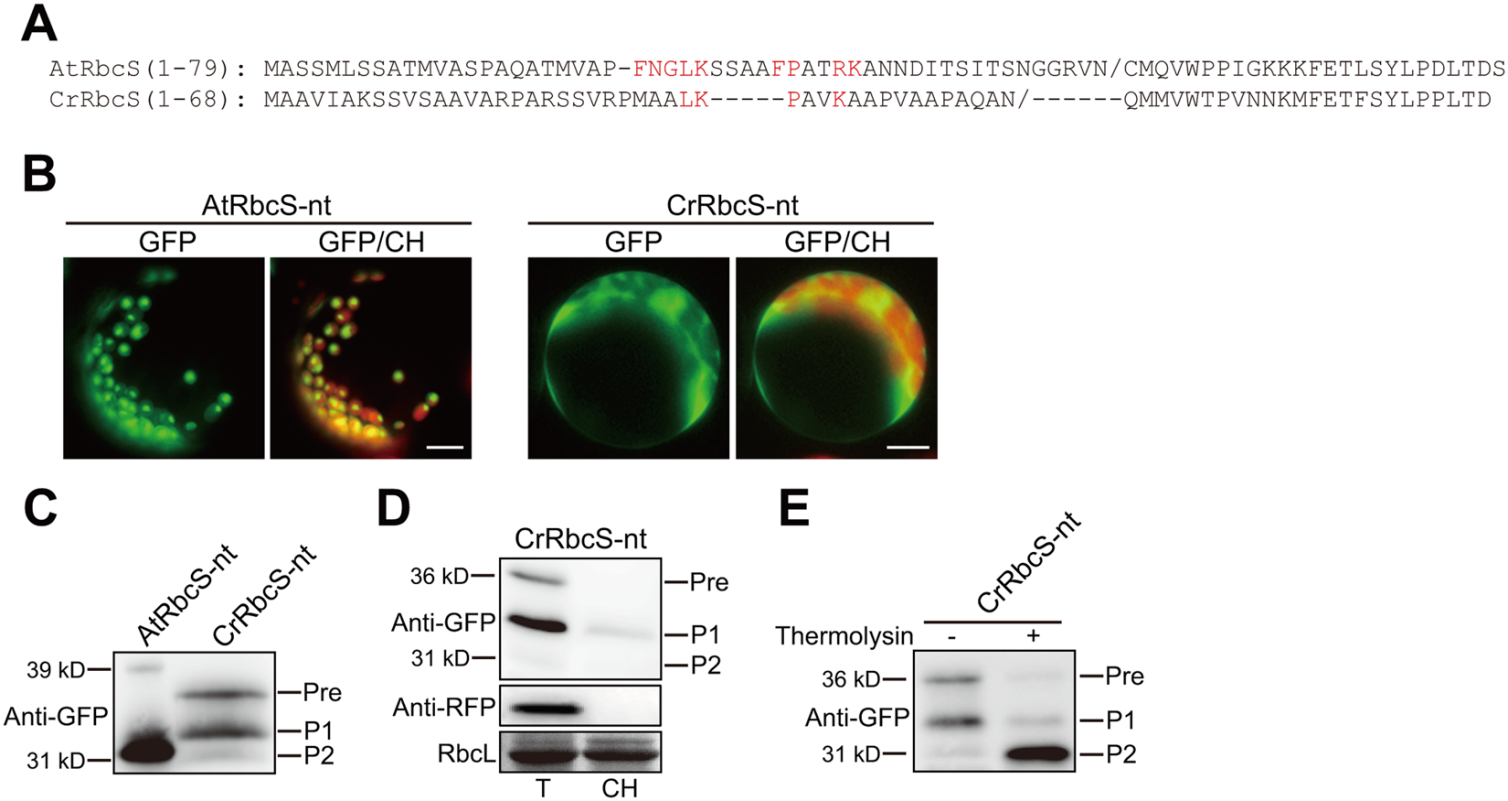
CrRbcS TP is not functional in Arabidopsis. (**A**) Sequences of RbcS TPs from Arabidopsis and *C. reinhardtii*. All constructs were fused to GFP. The cleavage sites predicted by ChloroP are indicated by ‘/’. (**B**) Localization of reporter proteins. Protoplasts from Arabidopsis plants were transformed with the indicated constructs, and GFP patterns were observed 12 h after transformation. Green, red, and yellow signals represent GFP, chlorophyll autofluorescence, and the overlap between green and red signals, respectively. Scale bar = 20 μm. (**C**) Western analysis of reporter proteins. Total protein extracts from transformed protoplasts were analyzed by western blotting with anti-GFP antibody. Pre, precursor form; P1, processed form 1; P2, processed form 2. (**D**) Isolation of chloroplasts from transformed protoplasts. Chloroplasts were isolated from protoplasts transformed with *CrRbcS-nt:GFP*. At 12 h after transformation, protoplasts were gently lysed and chloroplasts were isolated using a Percoll gradient. Total and chloroplast fractions were analyzed by western blotting with anti-GFP and anti-RFP antibodies. RFP was used as a control for cytosolic proteins. Rubisco complex large subunit (RbcL) stained with Coomassie brilliant blue was used as a loading control. T, total protein; CH, chloroplast fraction; Pre, precursor form; P1, processed form 1; P2, processed form 2. (**E**) Thermolysin sensitivity of CrRbcS-nt:GFP. Protoplasts transformed with *CrRbcS-nt:GFP* were gently lysed and treated with thermolysin. Protein extracts were analyzed by western blotting using anti-GFP antibody. Pre, precursor form; P1, processed form 1; P2, processed form 2.

Next, we examined whether sequence differences between algal and plant TPs reflect any functional differences using TPs of CrRbcS and AtRbcS as a model system (Fig. 1A). Previous studies showed that an N-terminal segment containing 60 to 80 amino acid residues is sufficient to efficiently deliver GFP into chloroplasts^6, 7, 10, 11^. An N-terminal 68-amino-acid segment of CrRbcS was fused to *GFP*, and the resulting construct, *CrRbcS-nt:GFP*, was introduced into Arabidopsis protoplasts. AtRbcS-nt:GFP, which consists of an N-terminal 79-amino-acid segment of AtRbcS fused to GFP^6^, was used as a control. As reported previously^6^, AtRbcS-nt:GFP exhibited a strong chloroplast localization pattern (Fig. 1B). By contrast, GFP expression was observed only in the cytosol, and not in chloroplasts, indicating that CrRbcS-nt:GFP was not imported into chloroplasts in Arabidopsis protoplasts (Fig. 1B). To confirm this, protein extracts from protoplasts were analyzed by western blotting using anti-GFP antibody. Consistent with the microscopy analysis, AtRbcS-nt:GFP was largely processed to a mature form with only a minimal amount of precursors. However, CrRbcS:GFP produced a different band pattern; the top band at the position of precursors (Pre) was equal with an intermediate (P1) in intensity alongside a minor processed form (P2) at the position of the mature form (Fig. 1C). Cellular localization of the two processed forms, P1 and P2, was analyzed by purifying chloroplasts. No P2 and only a small amount of P1 was copurified with chloroplasts, indicating that the processed forms were not imported into chloroplasts. Co-transformed cytosolic RFP was not detected in the chloroplast fraction, confirming successful fractionation (Fig. 1D). Furthermore, gently lysed protoplast extracts were treated with thermolysin, a protease that can degrade proteins in the cytosol and outer envelope proteins exposed to the cytosolic face. Both precursor and P1 were sensitive to thermolysin, confirming that they were not imported into chloroplasts (Fig. 1E). A previous study showed that properly folded GFP is resistant to thermolysin, which is the reason why a protein band almost identical to the P2 band in size was generated^6^ (Fig. 1E). These results strongly suggest that CrRbcS TP is not functional in Arabidopsis.

### The FP-RK motif of AtRbcS-nt rescues the defect of CrRbcS-nt in protein import into plant chloroplasts

**Figure 2:**
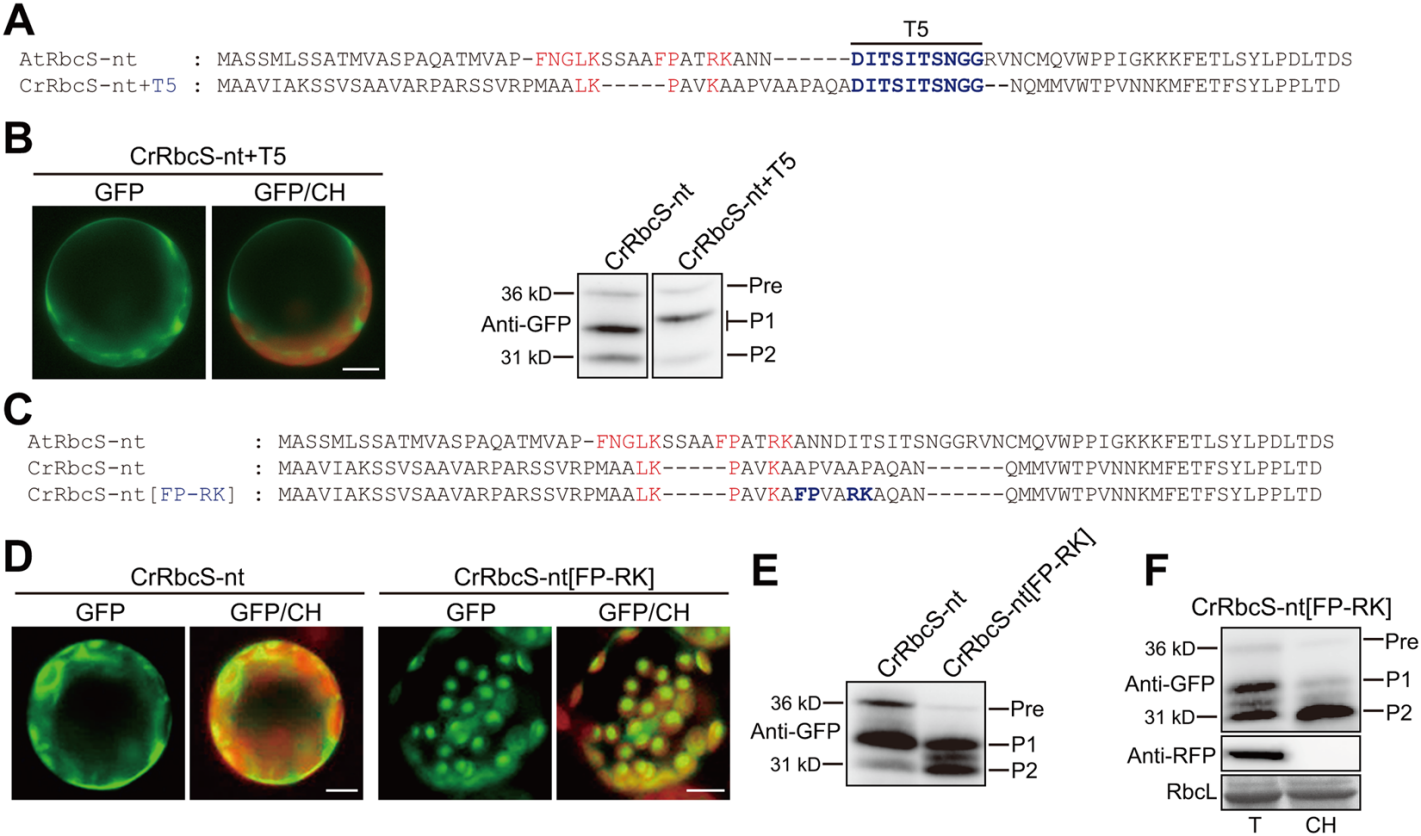
The FP-RK motif of AtRbcS-nt rescues the defect of CrRbcS-nt in protein import into plant chloroplasts. (**A** and **C**) Sequences of AtRbcS TP, CrRbcS TP, and modified CrRbcS TP. All constructs were fused to GFP. (**B** and **D**) Localization of reporter proteins. Protoplasts from Arabidopsis plants were transformed with the indicated constructs, and GFP patterns were observed 12 h after transformation. Green, red, and yellow signals represent GFP, chlorophyll autofluorescence, and the overlap between green and red signals, respectively. Scale bar = 20 μm. In the western blot image of Fig. 2B, both lanes were cropped from the different parts of the same gel (Fig. S3). (**E**) Western analysis of reporter proteins. Total protein extracts from transformed protoplasts were analyzed by western blotting with anti-GFP antibody. Pre, precursor form; P1, processed form 1; P2, processed form 2. (**F**) Isolation of chloroplasts from transformed protoplasts. Chloroplasts were isolated from protoplasts transformed with *CrRbcS-nt[FP-RK]:GFP*. At 12 h after transformation, protoplasts were gently lysed and chloroplasts were isolated using a Percoll gradient. Total and chloroplast fractions were analyzed by western blotting using anti-GFP and anti-RFP antibodies. RFP was used as a control for cytosolic proteins. Rubisco complex large subunit (RbcL) stained with Coomassie brilliant blue was used as a loading control. T, total protein; CH, chloroplast fraction; Pre, precursor form; P1, processed form 1; P2, processed form 2.

CrRbcS-nt was unable to deliver proteins into chloroplasts in plant cells, and we wished to determine what underlies this deficiency. The most conspicuous difference between the plant and algal TPs was that CrRbcS TP was shorter than AtRbcS TP. A previous study noted that internal deletions were detrimental to the activity of AtRbcS TP^6^. Here, we inserted the T5 segment of AtRbcS-nt into the corresponding region of CrRbcS to produce CrRbcS[1–68] + T5 because this segment was clearly absent in algal RbcS TP (Figs 2A and S1)^6^. The resulting construct was fused to GFP and introduced into protoplasts. CrRbcS[1–68] + T5 did not deliver proteins into chloroplasts (Fig. 2B), indicating that the elongated CrRbcS TP did not function as a TP in plant cells. Sequence alignment suggested that CrRbcS TP lacked some sequence motifs, such as FNGLK and FP-RK, which were crucial for protein import into chloroplasts in Arabidopsis (Fig. S1)^8, 12^. We asked whether introduction of these motifs to CrRbcS TP could rescue its activity and allow protein import into chloroplasts in Arabidopsis. We incorporated the sequence motif FP-RK, one of the most important Arabidopsis AtRbcS TP sequence motifs, at position 35 in CrRbcS-nt and examined the ability of GFP-fused CrRbcS[FP-RK] to deliver proteins into chloroplasts in Arabidopsis protoplasts (Fig. 2C). CrRbcS-nt[FP-RK]:GFP showed strong GFP signals in chloroplasts (Fig. 2D), indicating that the FP-RK motif greatly improved the protein delivery efficiency of CrRbcS TP in Arabidopsis. Protein import was assessed by western analysis using anti-GFP antibody. As with CrRbcS-nt:GFP, three protein forms (two processed and one precursor) were seen with CrRbcS-nt[FP-RK]:GFP. However, the band intensities seen with CrRbcS-nt[FP-RK]:GFP were markedly different. The intensity of the P2 band increased and concomitantly the intensity of the precursor band decreased in CrRbcS-nt[FP-RK]:GFP relative to CrRbcS-nt:GFP (Fig. 2E), indicating that protein delivery to chloroplasts was improved by inclusion of the FP-RK motif.

To further examine this, chloroplasts were isolated from protoplasts and protein extracts from the chloroplast fraction were analyzed by western blotting using anti-GFP antibody. Most of P2, and a small proportion of P1, copurified with chloroplasts, confirming that P2 was the stromal form of CrRbcS-[FP-RK]:GFP (Fig. 2F). P1 might be generated through proteolysis of the precursor form in the cytosol and be weakly associated with chloroplasts. These results confirmed that the FP/RK motif rescued the ability of CrRbcS-nt to import proteins into plant chloroplasts. RFP was included in transformations as a control for inadvertent copurification of cytosolic proteins. No RFP was detected in the chloroplast fraction, confirming that copurification was effective. RbcL was used as a control for equal loading and chloroplast isolation (Fig. 2F). Together, these results indicate that 1) CrRbcS-nt contains some, but not all, of the critical sequence motifs required for protein import into plant chloroplasts, and that 2) the FP-RK motif can complement missing motifs in CrRbcS-nt and allow protein import to occur.

### The position of the FP-RK motif in CrRbcS TP is critical for its function in protein import into plant chloroplasts

**Figure 3:**
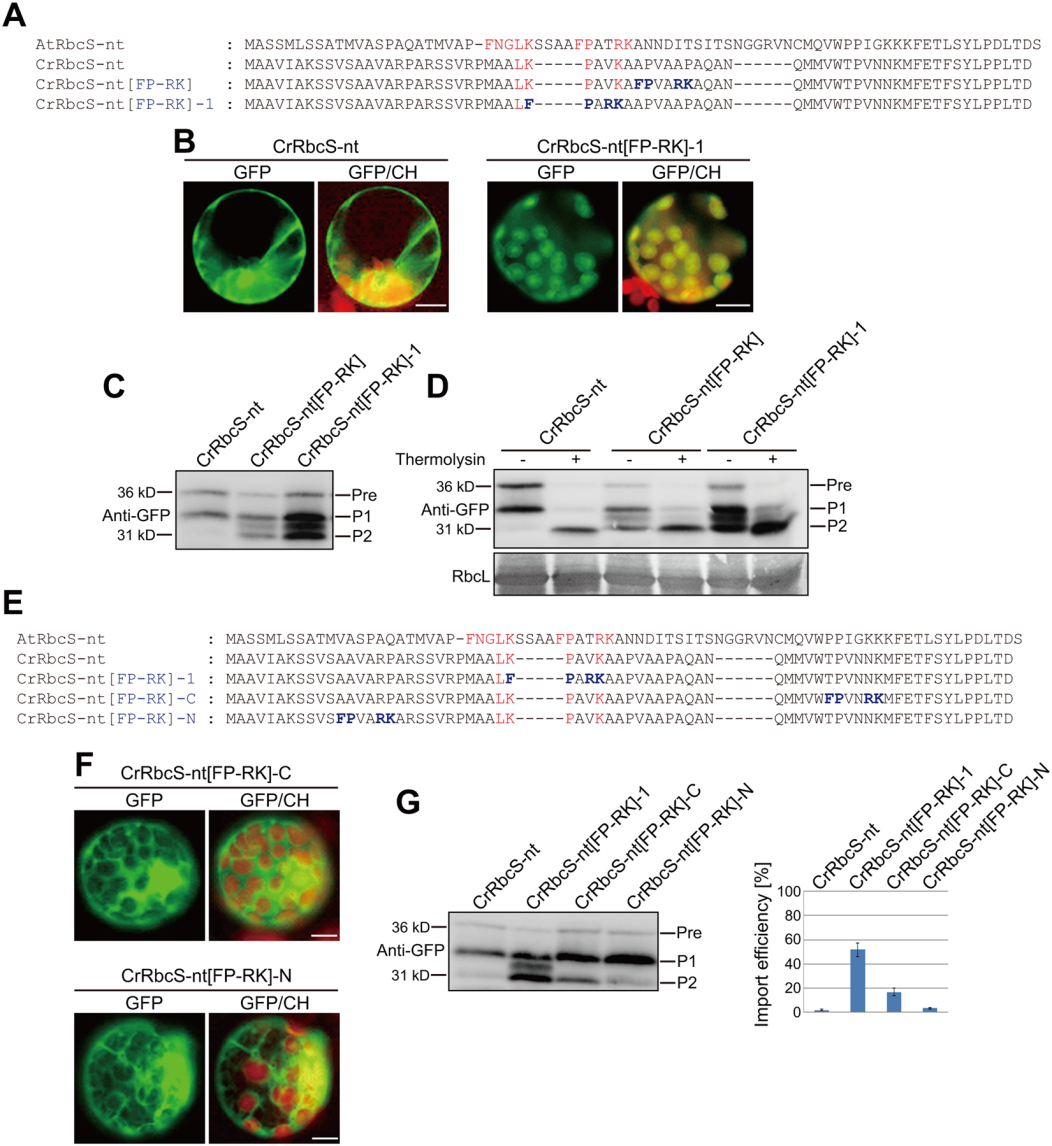
The position of the FP-RK motif in CrRbcS TP is critical for its function in protein import into plant chloroplasts. (**A** and **E**) Sequences of AtRbcS TP, CrRbcS TP, and modified CrRbcS TPs. All constructs were fused to GFP. (**B** and **F**) Localization of reporter proteins. Protoplasts from Arabidopsis plants were transformed with the indicated constructs, and GFP patterns were observed 12 h after transformation. Green, red, and yellow signals represent GFP, autofluorescence of chlorophyll, and the overlap between green and red signals, respectively. Scale bar = 20 μm. (**C** and **G**) Western analysis of reporter proteins. Total protein extracts from transformed protoplasts were analyzed by western blotting with anti-GFP antibody. Pre, precursor form; P1, processed form 1; P2, processed form 2. Signal intensity of protein bands was measured using LAS3000 imager (FUJI FILM) software, and import efficiency was defined as the amount of P2 relative to the total amount of expressed protein. Three independent transformation experiments were performed, and the data represent means with standard deviation (SD). (**D**) Thermolysin sensitivity of reporter proteins. Protoplasts transformed with the indicated constructs were gently lysed and treated with thermolysin. Protein extracts were analyzed by western blotting using anti-GFP antibody. Pre, precursor form; P1, processed form 1; P2, processed form 2.

The FP-RK motif is predominantly located in the middle of RbcS TPs (Fig. S1). Thus, we tested whether FP-RK function was dependent on position within the TP. First, an alternative FP-RK construct (*CrRbcS-nt[FP-RK]-1*) was created in which the FP-RK motif was moved six amino acids towards the N-terminus relative to its position in CrRbcS-nt[FP-RK] (Fig. 3A). In this position, CrRbcS-nt contained P and R residues equivalent to the second and third residues, respectively, of the FP-RK motif. The construct was fused to GFP and introduced into Arabidopsis protoplasts. CrRbcS-nt[FP-RK]-1 also efficiently delivered GFP into chloroplasts (Fig. 3B), indicating that the minor change in the location of FP-RK in the TP did not affect its protein delivery function. Protein extracts from protoplasts were again analyzed by western blotting using anti-GFP antibody. As with CrRbcS-nt[FP-RK], two processed protein forms and a small proportion of precursor were observed with CrRbcS-nt[FP-RK]-1 (Fig. 3C). Import of P2 into chloroplasts was also assessed by treating gently lysed protoplasts with thermolysin. P2 was protected from thermolysin, whereas the precursor and P1 were largely subjected to proteolytic degradation, confirming that P2 was imported into chloroplasts (Fig. 3D).

Two additional constructs were generated, namely, *CrRbcS-nt[FP-RK]-N* and *CrRbcS-nt[FP-RK]-C*, with insertions of the FP-RK motif at positions 12 and 50 of CrRbcS-nt, respectively (Fig. 3E). The resulting TPs were fused to GFP and introduced into protoplasts. Severe defects in chloroplast targeting were seen with both constructs, and cytosolic GFP was observed (Fig. 3F). Protein extracts from protoplasts were analyzed by western blotting using anti-GFP antibody. By contrast with CrRbcS-nt[FP-RK], both CrRbcS-nt[FP-RK]-N and CrRbcS-nt[FP-RK]-C produced largely the P1 protein form and only small proportions of the precursor and P2 (Fig. 3G). Import efficiencies, as expressed by the amount of P2 relative to total expressed proteins, were only 4% and 18% for CrRbcS-nt[FP-RK]-N and CrRbcS-nt[FP-RK]-C, respectively, compared with 50% for CrRbcS-nt[FP-RK]. This indicated that the position of the FP-RK motif was critical for protein import function.

**Figure 4:**
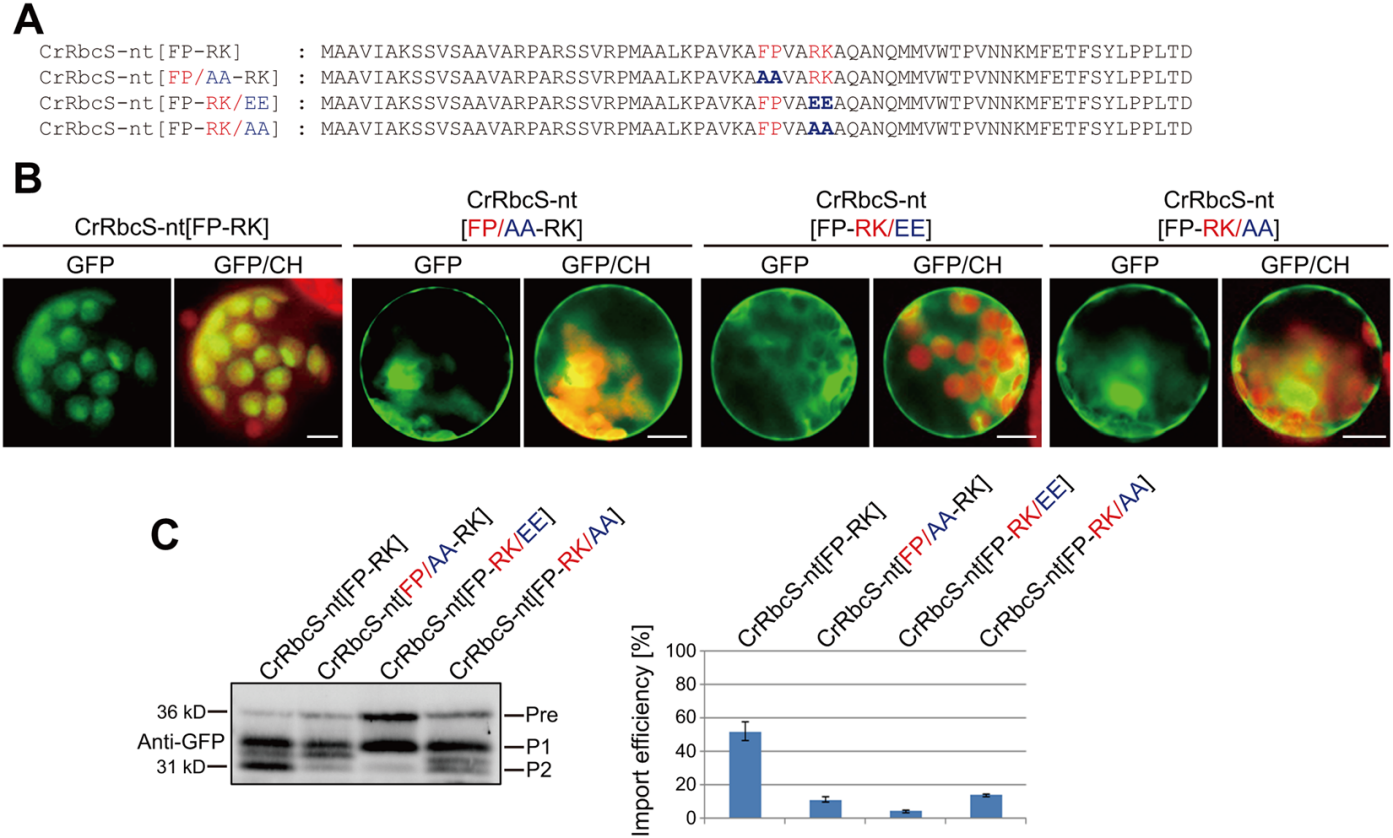
The FP-RK motif is critical in CrRbcS-nt[FP-RK] for protein import into chloroplasts. (**A**) Sequences of CrRbcS-nt[FP-RK] and its substitution mutants. All constructs were fused to GFP. (**B**) Localization of reporter proteins. Protoplasts from Arabidopsis plants were transformed with the indicated constructs, and GFP patterns were observed 12 h after transformation. Green, red, and yellow signals represent GFP, chlorophyll autofluorescence, and the overlap between green and red signals, respectively. Scale bar = 20 μm. (**C**) Western analysis of the reporter proteins. Total protein extracts from transformed protoplasts were analyzed by western blotting using anti-GFP antibody. Pre, precursor form; P1, processed form 1; P2, processed form 2. Signal intensity of protein bands was measured using LAS3000 imager (FUJI FILM) software, and import efficiency was defined as described in Fig. 3G. Error bar = SD (n = 3).

The results shown in Fig. 3 strongly suggested that the FP-RK motif in the middle position complemented the defect of CrRbcS-nt in delivering protein import into chloroplasts in Arabidopsis. However, other possibilities could not be excluded, such as improvement of CrRbcS-nt import efficiency by inserting additional amino acids at that position. The FP-RK motif contains the aromatic amino acid phenylalanine (F) and positively charged amino acids arginine (R) and lysine (K), which are found in most TPs. To examine the importance of the conserved FP-RK motif in chloroplast import, the FP or RK residues of the FP-RK motif were substituted with alanine or glutamic acid and import efficiency was assessed (Fig. 4A). First, FP or RK was substituted with two alanines to produce CrRbcS-nt[FP/AA-RK] and CrRbcS-nt[FP-RK/AA], respectively. The resulting constructs were introduced into protoplasts after fusion to GFP. The alanine-substituted mutants exhibited strong GFP signals in the cytosol with concomitant decreases in GFP signals in chloroplasts, indicating that both FP and RK residues in CrRbcS-nt[FP-RK] were crucial for protein import into chloroplasts (Fig. 4B). Next, the positively charged R and K residues were replaced with the negatively charged residue glutamic acid (E), which is under-represented in TPs. CrRbcS-nt[FP-RK/EE] also showed a strong cytosolic GFP signal (Fig. 4B).

Protein extracts from protoplasts were analyzed by western blotting using anti-GFP antibody. In contrast with CrRbcS-nt[FP-RK], alanine- or glutamic acid-substituted mutants showed significant decreases in P2 (Fig. 4C). The import efficiencies of CrRbcS-nt[FP/AA-RK], CrRbcS-nt[FP-RK/AA], and CrRbcS-nt[FP-RK/EE] were approximately 15%, 18%, and 5%, respectively, confirming that the specific FP-RK motif was critical for the chloroplast protein import function of CrRbcS-nt[FP-RK].

### Two critical AtRbcS sequence motifs, FNGLK and FR-PK, improve the chloroplast protein import ability of CrRbcS-nt

**Figure 5:**
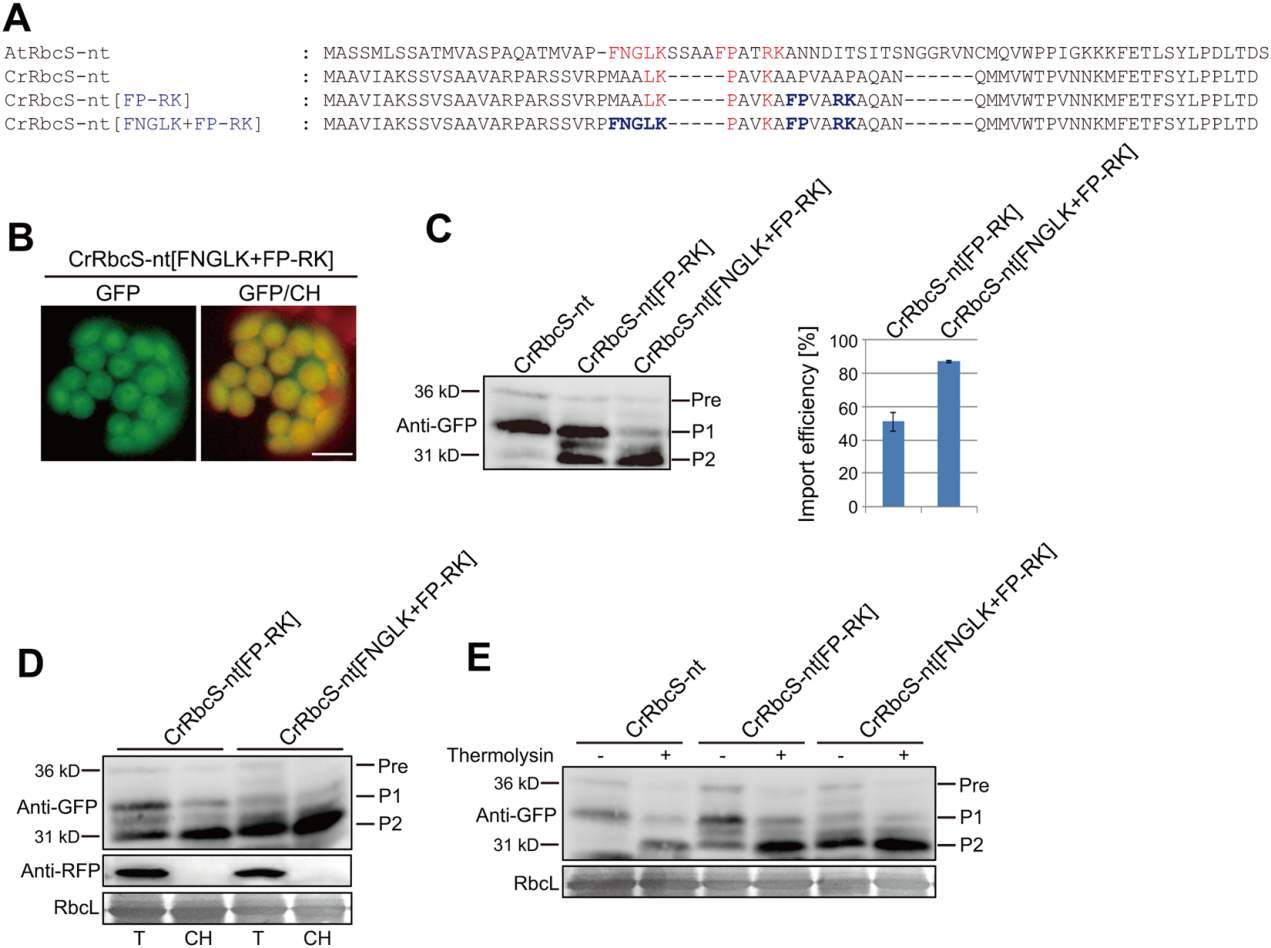
Two sequence motifs, FNGLK and FR-PK, of AtRbcS TP further improve the ability of CrRbcS-nt to import proteins into plant chloroplasts. (**A**) Sequences of AtRbcS TP, CrRbcS TP, and modified CrRbcS TPs. All constructs were fused to GFP. (**B**) Localization of reporter proteins. Protoplasts from Arabidopsis plants were transformed with the indicated constructs, and GFP patterns were observed 12 h after transformation. Green, red, and yellow signals represent GFP, chlorophyll autofluorescence, and the overlap between green and red signals, respectively. Scale bar = 20 μm. (**C**) Western analysis of the reporter proteins. Total protein extracts from transformed protoplasts were analyzed by western blotting using anti-GFP antibody. Pre, precursor form; P1, processed form 1; P2, processed form 2. Signal intensity of protein bands was measured using LAS3000 imager (FUJI FILM) software, and import efficiency was defined as described in Fig. 3G. Error bar = SD (n = 3). (**D**) Isolation of chloroplasts from transformed protoplasts. Chloroplasts were isolated from the protoplasts transformed with the indicated constructs. At 12 h after transformation, protoplasts were gently lysed and chloroplasts were isolated using a Percoll gradient. Total and chloroplast fractions were analyzed by western blotting using anti-GFP and anti-RFP antibodies. RFP was used as a control for cytosolic proteins. Rubisco complex large subunit (RbcL) stained with Coomassie brilliant blue was used as a loading control. T, total protein; CH, chloroplast fraction; Pre, precursor form; P1, processed form 1; P2, processed form 2. (**E**) Thermolysin sensitivity of reporter proteins. Protoplasts transformed with the indicated constructs were gently lysed and treated with thermolysin. Protein extracts were analyzed by western blotting using anti-GFP antibody. Pre, precursor form; P1, processed form 1; P2, processed form 2.

The sequence motif FNGLK is also critical for chloroplast protein import mediated by AtRbcS TP^8, 12^. This motif was tested for its ability to improve transport by CrRbcS-nt in Arabidopsis. FNGLK was introduced into CrRbcS-nt[FP-RK] to give CrRbcS-nt[FNGLK + FP-RK] (Fig. 5A). The resulting construct was fused to GFP and introduced into protoplasts, and GFP signals were strongly detected in the chloroplasts (Fig. 5B). Protoplast proteins were analyzed by western blotting using anti-GFP antibody. CrRbcS-nt[FNGLK + FP-RK] produced the same three protein bands as were seen with CrRbcS-nt[FP-RK], but band pattern and intensity differed. CrRbcS-nt[FNGLK + FP-RK] largely produced the P2 protein, with only small proportions of P1 and the precursor, indicating that the FNGLK motif further increased import efficiency compared with the FP-RK motif alone. Import efficiency of CrRbcS-nt[FNGLK + FP-RK] was close to 90%, very similar to that of AtRbcS-nt in Arabidopsis protoplasts (Fig. 5C). To confirm import, extracts of purified chloroplasts and protoplast extracts that had been treated with thermolysin were analyzed by western blotting using anti-GFP antibody. As before, P2 copurified with chloroplasts and was resistant to thermolysin treatment, confirming that P2 was imported into chloroplasts (Fig. 5D and E).

### CrRbcS-nt[FP-RK] and CrRbcS-nt[FNGLK + FP-RK] efficiently deliver proteins into *C. reinhardtii* chloroplasts

**Figure 6:**
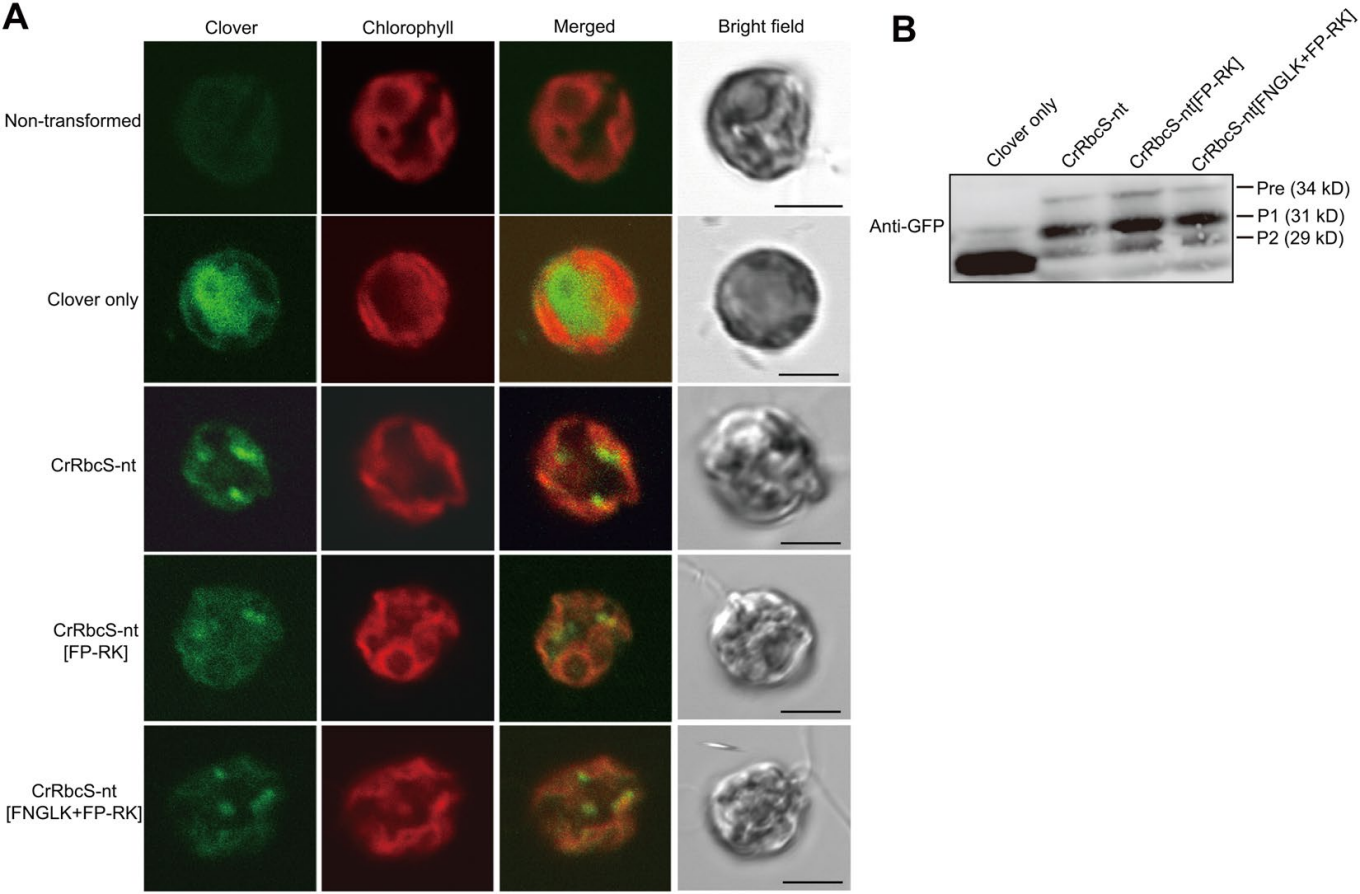
CrRbcS-nt[FP-RK] and CrRbcS-nt[FNGLK + FP-RK] efficiently deliver protein into the chloroplast in *C. reinhardtii*. (**A**) Localization of reporter proteins. *C. reinhardtii* (wild-type strain CC-503 cw92 mt+) was transformed with the indicated constructs, and Clover patterns were examined using a confocal laser scanning microscope. Green, red, and yellow signals represent Clover, chlorophyll autofluorescence, and the overlap between green and red signals, respectively. Scale bar = 5 μm. (**B**) Western analysis of the reporter proteins. Total protein extracts from transformed *C. reinhardtii* were analyzed by western blotting using anti-GFP antibody. Pre, precursor form; P1, processed form 1; P2, processed form 2.

Next, the behavior of modified CrRbcS-nt constructs was examined in *C. reinhardtii*. First, the *CrRbcS(1–68):Clover* (a GFP variant) construct was generated and transformed into *C. reinhardtii* (Fig. 6A). Transgenic *C. reinhardtii* expressing Clover only was also generated as a control. Transgenic algae were examined using a laser scanning confocal microscope. Transgenic *C. reinhardtii* transformed with *Clover only* exhibited Clover signal in the cytosol (Fig. 6A), but CrRbcS-nt:Clover distribution differed to that of Clover only. CrRbcS-nt:Clover was observed as punctate as well as diffuse signals that overlapped the chloroplast, indicating that CrRbcS-nt:Clover was targeted to chloroplasts.

*CrRbcS-nt[FP-RK]:Clover* and *CrRbcS-nt[FNGLK + FP-RK]:Clover* were then generated and transformed into *C. reinhardtii* (Fig. 6A). Multiple transgenic *C. reinhardtii* lines were isolated and used for examination of Clover distribution. Both CrRbcS-nt[FP-RK]:Clover and CrRbcS-nt[FNGLK + FP-RK] were imported into chloroplasts (Fig. 6A). These results indicated that insertion of the FP-RK motif alone or together with the FNGLK motif did not adversely affect the ability of CrRbcS-nt to deliver proteins into the chloroplast in *C. reinhardtii*.

Protein import was confirmed by western analysis of protein extracts from transgenic lines of *C. reinhardtii* using an anti-GFP antibody. The three transgenic lines harboring wild-type or hybrid *CrRbcS-nt* constructs all exhibited a strong band at 31 kDa alongside two minor bands at 29 and 34 kDa. The 34 kDa and 31 kDa forms corresponded to precursor and processed mature forms, respectively, and the 29 kDa band may have represented a further processed form. By contrast, transgenic lines containing Clover alone produced a single band at 29 kDa, the predicted molecular weight of Clover (Fig. 6B). These results confirmed that CrRbcS-nt with additional motifs from AtRbcS-nt was efficiently imported into chloroplasts in *C. reinhardtii*.

### CrRbcS-nt contains a sequence motif that is functional for protein import into plant chloroplasts

**Figure 7:**
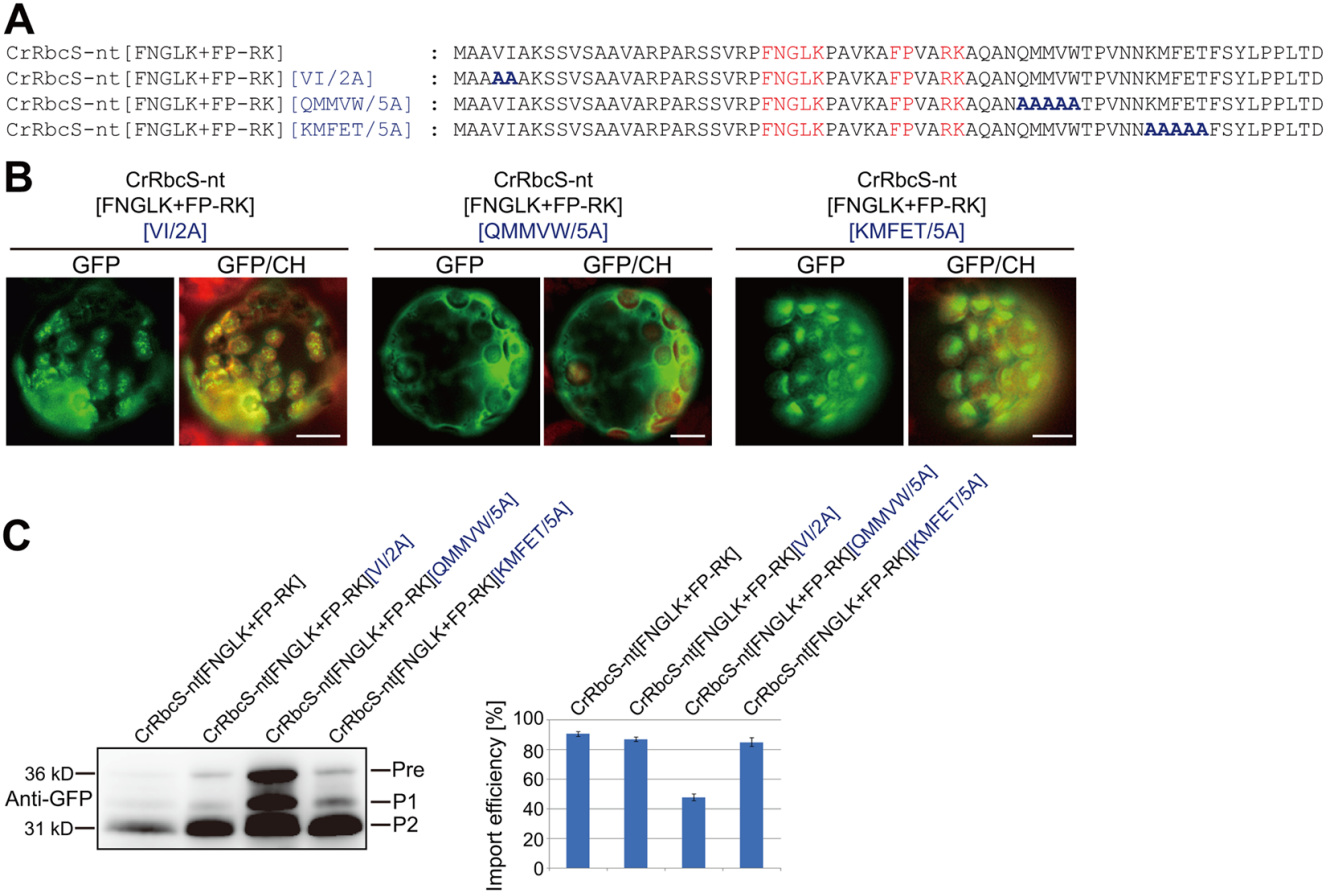
CrRbcS-nt contains a sequence motif that is functional in protein import into plant chloroplasts. (**A**) Sequences of CrRbcS-nt[FNGLK + FP-RK] and substitution mutants. All constructs were fused to GFP. (**B**) Localization of reporter proteins. Protoplasts from Arabidopsis plants were transformed with the indicated constructs, and GFP patterns were observed 12 h after transformation. Green, red, and yellow signals represent GFP, chlorophyll autofluorescence, and the overlap between green and red signals, respectively. Scale bar = 20 μm. (**C**) Western analysis of the reporter proteins. Total protein extracts from transformed protoplasts were analyzed by western blotting using anti-GFP antibody. Pre, precursor form; P1, processed form 1; P2, processed form 2. Signal intensity of protein bands was measured using LAS3000 imager (FUJI FILM) software, and import efficiency was defined as described in Fig. 3G. Error bar = SD (n = 3).

Multiple sequence motifs are required for efficient protein import into chloroplasts in plants. The results showing that CrRbcS-nt[FP-RK] could deliver proteins into plant chloroplasts prompted us to examine whether CrRbcS-nt contained additional sequence motifs other than FNGLK and FP-RK that contributed to delivering proteins to plant chloroplasts. We selected three regions in the CrRbcS-nt[FNGLK + FP-RK] and substituted with alanines (Fig. 7A). The corresponding regions in AtRbcS TP contain functional sequence motifs^6^. Of these mutants, VI/2 A and KKFET/5 A showed only minor or no defects in protein import (Fig. 7B and C). However, protein import into chloroplasts was greatly impaired with alanine substitution of QMMVW (Fig. 7B and C), indicating that the QMMVW motif of CrRbcS-nt was functional in plants^6^.

## Discussion

In this study, we showed that CrRbcS TP did not support protein import into chloroplasts in Arabidopsis. This is in contrast to the observation that the low-CO_2_-inducible *C. reinhardtii* proteins LCIA, LCIB and LCIC were successfully imported into chloroplasts when expressed in tobacco^15^. One possible explanation for the difference in capacity of RbcS TPs between Arabidopsis and *C. reinhardtii* in delivering protein into chloroplasts in plants is that RbcS TPs gained additional motifs for protein import in plant cells during evolution. In fact, plant TPs are longer than those in algae. The central region appears to be inserted between conserved N- and C-terminal regions of algal TPs to give rise to plant TPs. Consistent with this idea, critical sequence motifs were identified in the central region of AtRbcS TP^6, 7, 12^. Moreover, when the FP-RK motif, one of the sequence motifs in the central region, was introduced into CrRbcS-nt, the mutant TP, CrRbcS-nt[FP-RK], was able to support protein import into chloroplasts in Arabidopsis. Furthermore, the FNGLK motif, another sequence motif in the central region, further improved the protein import efficiency of CrRbcS TP. These results suggest that these plant motifs function together with sequence motifs in CrRbcS TP to efficiently deliver protein into chloroplasts in plants. One of the CrRbcS motifs that function as a critical sequence motif in plants was the QMMVW motif at the C-terminal region of CrRbcS-nt. The CrRbcS QMMVW motif resembles the CMQVW motif of AtRbcS TP^6^. Thus, these results support gaining of sequence motifs in plant RbcS TPs during evolution. Although we favor this idea, we cannot exclude the possibility that CrRbcS TPs might have lost these sequence motifs from the unknown ancestral RbcS TP because they were not essential in protein import into *C. reinhardtii*. Consistent with this idea, the presence and absence of the sequence motifs, FP-RK and FNGLK, did not affect not only protein import into chloroplasts in *C. reinhardtii* but also preprotein processing.

Another important finding in this study was that the critical sequence motifs needed to be placed in the center of the CrRbcS TP to be functional. This corresponded with the original source location of the two sequence motifs in the center of the RbcS TP. These results suggest that the critical sequence motifs require correct placement to function, consistent with results from previous studies^8, 10, 16^. First, Lee *et al*.^8^ showed that hybrid TPs generated by swapping domains between two different types of TPs (RbcS and Cab TPs) could support protein import into chloroplasts as efficiently as the wild-type TPs when the interchanged domains were appropriately fused to one another. Correspondingly, the sequence motifs become non-functional when positioned inappropriately in the domain-swapping mutants. Second, Rolland *et al*.^10^ showed that both cTP and MPL (membrane protein leader) located between cTP and the first TMD (transmembrane domain) are required for targeting to inner envelope membrane of chloroplasts. Interestingly, MPL was interchangeable between different inner envelope proteins. Moreover, when the primary sequence of MPL possessing asymmetric charge distribution was inverted, the functionality of TP was lost, suggesting that the position of charged residues is critical for chloroplast targeting. The factors underlying the positional dependency of TP sequence motifs remain to be determined, but may be linked to motif functions. The entire import process may include multiple distinct steps including cytosolic sorting, binding to receptors at the chloroplast surface, translocation through the two envelope membranes, and pulling the protein into the stroma^3, 5, 17^. Thus, the sequence motifs in TPs are likely recognized by factors in the cytosol, components of translocons at the outer and inner membranes, and chaperones in the stroma during protein import into the chloroplasts^5, 17^.

Although almost all the translocon components of the translocon machinery are conserved between *C. reinhardtii* and Arabidopsis^18, 19^, the composition of Toc/Tic components may differ between algae and plants. Toc/Tic translocons may be more complex in higher plants than in *C. reinhardtii*: multiple isoforms of Toc/Tic components are present in Arabidopsis, whereas the *C. reinhardtii* translocon contains relatively few isoforms of simpler structure^19^. Another possibility is that low sequence homology between Arabidopsis and Chlamydomonas Toc/Tic complexes affects the binding affinity for TPs. AtRbcS TP contains the FGLK motif that binds to Toc33/Toc34^12^. The FP/RK motif is closely related to the FGLK motif and may also bind to Toc33/Toc34. CrRbcS TP does not contain the FP/RK motif and may not bind efficiently to Arabidopsis Toc33/Toc34, which would underlie the failure of CrRbcS TP in delivering protein into Arabidopsis chloroplasts. However, in CrRbcS TP, the sequence motif for Toc33/34-binding may differ from the Arabidopsis motif. Indeed, sequence motifs are highly flexible and can be replaced by other motifs of completely different amino acid sequence^8^. For example, RbcS TP contains functionally redundant motifs that have no sequence similarity^6^, and CrRbcS TP may thus contain an alternative motif for binding to Toc33/Toc34 in *C. reinhardtii*. Alternative possibilities are that a specific motif is not required for CrRbcS TP binding to Toc33/Toc34, or that binding to Toc33/Toc34 is less critical for protein import into chloroplasts in *C. reinhardtii* than in higher plants because in the alga, the chloroplast occupies most of the cellular space.

What would have been the driving force underpinning acquisition of new sequence motifs in TPs for delivery of proteins to chloroplasts in higher plants? One prominent change that occurred during plant evolution was the increase in cell size. TPs of chloroplast proteins would need to bind to chloroplast surface receptors more efficiently to facilitate effective protein import in a larger cellular environment. This might have been achieved by acquiring additional sequence motifs in TPs and additional import receptors at the surface of chloroplasts. The FGLK motif found in plant TPs binds to Toc34 and possibly other Toc components of the Toc/Tic complex^12^. In the case of Toc34, two isoforms (Toc33 and Toc34) are present in Arabidopsis^20^. Likewise, multiple Toc159 isoforms including Toc159, Toc132, Toc120, and Toc90 are found in plants^21–23^. Consistent with this, GFP fused to CrRbcS TP largely remained in the cytosol in Arabidopsis protoplasts, indicating inefficient binding to chloroplast receptors. Although this hypothesis remains to be tested, multiple receptors and multiple binding motifs in TPs might have facilitated highly efficient binding and protein import into chloroplasts despite the larger relative size of the cell.

## Methods

### Plant Materials and Cultivation

*Arabidopsis thaliana* (Columbia-0 ecotype) was grown in a growth chamber at 22–23 °C with a 16 h light/8 h dark cycle on Gamborg B5 agar plates. Leaf tissues were harvested from 2 to 3-week-old plants.

### Plasmid Construction

A DNA fragment encoding the N-terminal 68-amino-acid fragment of CrRbcS, termed *CrRbcS-nt*, was generated by three successive PCR reactions with three forward primers in which GC contents were modified (Primer 1, Primer 2, and then Primer 3) and a reverse primer (nosT-B) with *AtRbcS-nt:GFP* as template (Table S2). Substitution mutants used in this study were generated by PCR as described previously^6^. Primer sequences used for generation of mutant constructs are shown in Table S2.

### PEG-Mediated Transformation of Arabidopsis Protoplasts

Plasmid DNA used for PEG (polyethylene glycol)-mediated transformation was purified using a Qiagen MIDI kit (Qiagen). Plasmid DNA was transformed into Arabidopsis protoplasts by the PEG-mediated transformation method as described in detail previously^24, 25^. Briefly, Arabidopsis leaf tissues were incubated in the enzyme solution containing cellulose and macerozyme with gentle agitation overnight. Protoplast solution was passed through the 100-μm mesh to remove debris. Harvested protoplasts were loaded onto 21% sucrose solution followed by centrifugation at 730 rpm (98 × *g*) for 10 min. The intact protoplasts were isolated from the top and interface fractions and used for PEG-mediated transformation. Plasmid DNA (10 μg) was transformed into protoplasts by PEG-mediated transformation.

### Fluorescence Microscopy and Immunoblotting

Images of subcellular localization in transformed protoplasts were acquired as described previously^25^. Images were obtained using a cooled CCD camera and a Zeiss Axioplan-fluorescence microscope at 400 x magnification. The filter sets used were XF116 (exciter, 474AF20; dichroic, 500DRLP; emitter, 510AF23) and XF137 (exciter, 540AF30; dichroic, 570DRLP; emitter, 585ALP) (Omega, Inc., Brattleboro, VT) for green fluorescent protein and autofluorescence of chlorophyll, respectively. Adobe Photoshop software was used to equally balance images for brightness and contrast and to process image data. Protoplasts were resuspended in lysis buffer (50 mM Tris-HCl, pH 7.5, 150 mM NaCl, 1 mM EDTA, 1% Triton X-100, and 1 × protease inhibitor cocktail) and lysed by brief sonication. Cell lysates were centrifuged at 3,000 × g at 4 °C for 10 min to remove cell debris. Protein extracts were separated by SDS-PAGE and analyzed by western blotting using appropriate antibodies. Anti-GFP antibody was purchased from Clontech (Cat. #632381). Proteins were visualized using enhanced chemiluminescence (ECL kit; Amersham Pharmacia Biotech), and images were obtained using a LAS 3000 image capture system (FUJIFILM). Immunoblots were quantified by measuring the intensity of protein bands using LAS 3000 imager software (FUJIFILM).

To estimate chloroplast import efficiency, the intensity of the P2 form was divided by the sum of total expressed proteins. Three independent transformation experiments were performed. Calculations were performed using Microsoft Excel software.

### Chloroplast Isolation

Chloroplasts were isolated from protoplasts using a Percoll gradient^7^. Briefly, transformed protoplasts were gently lysed in ice-cold HMS buffer. Lysed protoplasts were overlaid on top of step-wise gradient consisting of 20% and 80% of percoll, and centrifuged at 3000 rpm for 5 min. The intact chloroplasts at the interface were harvested as a chloroplast (CH) fraction.

### *C. reinhardtii* Strains and Culture Conditions

*C. reinhardtii* wild-type strain CC-503 cw92 mt+ was obtained from the Chlamydomonas Resource Center (USA). Cells were cultured in a 500 ml Erlenmeyer flask containing 200 ml Tris-acetate-phosphate (TAP) medium at 23 °C under continuous light (75 µmol photons/m^2^/s) in a shaker.

### Generation of Transgenic *C. reinhardtii* and Subcellular Localization of CrRbcS TP in *C. reinhardtii*

*CrRbcS-nt* was inserted into the pOpt_Clover_Paro vector^26, 27^ using *Nsi*I and *Bgl*II sites to produce pOpt-CrRbcS-Clover-Paro. *C. reinhardtii* wild-type strain CC-503 cw92 mt+ was transformed with *pOpt-CrRbcS-Clover-Paro* by electroporation^28^. Briefly, Chlamydomonas cells grown to mid-log phase in TAP medium were subject to centrifugation at 1,800 × g at 16 °C for 5 min. Chlamydomonas cells resuspended with TAP medium containing 60 mM sucrose were transformed with 1 μg of plasmid DNA by electroporation, followed by recovery in TAP medium containing 60 mM sucrose for 16 h. Transformed Chlamydomonas cells were subjected to centrifugation at 1,800 × g at 16 °C for 5 min, followed by selection on the paromomycin-containing medium.

Transformed cells were observed using a laser scanning confocal microscope (Olympus Fluo View™FV1000). Transformed cells were excited at 488 nm. Clover fluorescence was simultaneously collected at 500–530 nm, and chlorophyll fluorescence was collected at 650–700 nm.

### Protein Extraction from *C. reinhardtii* and Immunoblotting

Transformed Chlamydomonas cultures were grown in 5 ml TAP medium until late log phase, collected by centrifugation at 1,800 × g, and resuspended in a buffer containing 50 mM Tris-HCl, pH 7.5, 1 mM EDTA, 150 mM NaCl, 1% SDS, and 1 × protease inhibitor cocktail. Cells were lysed by sonication and centrifuged at 3,000 × g for 10 min. Protein extracts were collected, and protein concentration was measured using a Bradford assay (Bio-Rad, Hercules, CA USA). Proteins (10 µg per sample) were separated by 10% SDS-PAGE and analyzed by western blotting using an anti-GFP antibody.

### Data availability statement

All data generated or analyzed during this study are included in this published article (and its Supplementary Information files).

## Electronic supplementary material

Supplementary information
