# Supplementary material for: Evolution of rubisco complex small subunit transit peptides from algae to plants
Source: Sci Rep. 2017 Aug 24;7:9279. doi: 10.1038/s41598-017-09473-x (PMC5571161; doi:10.1038/s41598-017-09473-x)
Supplement: Supplementary file 1 — Supplementary information [file 41598_2017_9473_MOESM1_ESM.pdf]

Supplementary information

Evolution of rubisco complex small subunit transit peptides from algae to plants

Md. Abdur Razzak[1], Dong Wook Lee[1], Yun-Joo Yoo and Inhwan Hwang[*]

Division of Integrative Biosciences and Biotechnology, Pohang University of Science and Technology, Pohang, 37673, Korea

[1]The first two authors contributed equally to this work

[*]Corresponding author: To whom correspondence should be addressed

E-mail: ihhwang@postech.ac.kr

Tel: 82-54-279-2128

Fax: 82-54-279-8159

Figure S1. Multiple sequence alignments of RbcS TPs from several species.

RbcS TP sequences (containing cTP and 10 amino acids from mature region) from algae and land plants were subjected to multiple sequence alignments using Clustal Omega. The FGLK and FP-RK motifs are highlighted in yellow. The T5 segment is highlighted in green.

Figure S2. Phylogenetic analysis of RbcS TPs from algae and higher plants.

The maximum likelihood phylogeny of RbcS TP sequences from 33 algal and 83 land plants. The phylogenetic tree was generated using MEGA5[1] with 1000 bootstrap replicates.

Figure S3. Original gel scan.

Table S1. The information for species used for multiple sequence alignments of RbcS TPs.

The species shown were selected from BLASTP analysis using CrRbcS(1-68) sequence as a query.

Table S2. Primer sequences used to generate the constructs in this study.

Figure S1. Multiple sequence alignments of RbcS TPs from various species.

(Algae)

```
P16131             MATTMLNRS---VIV------------NKEVAKTPNFPRATKNNKGFASNAAVQKCRDMMVWQPFNN  52
P16134             MASIMMNKS---VVL------------SKECAKPLATPKVTLNKRGFATTIA-TKNREMMVWQPFNN  51
P26985             -----MSFA---TT--------------NKTIVPCATTKQIVRPRFLSNGTISKSRAMMVWEPFNN  44
Q68BK3             MASALA-FT---PAL------------VRPTGVKTT--VASKKNVFAVSNSTIKNTSAFMVWTPVNN  49
BAD42333.1         MASTIA-IS---TAA------------VRIAPITKVNAASTKARTAFVSNGTVKKTTAMLVWTPINN  51
BAD42334.1         MASTIA-FS---TAA------------VRVAPITKVNATSTKARTAFVSNGTVKKTTAMLVWTPINN  51
C7FF10             MACTMAAIA---PVA------------VRPVAG--LKQAKNTSAARTVSNGSIQKTTAMQVWTPINN  50
E1Z3C8             MASTMAAIA---PVA------------VRPMASTPLKQAKNTFAARTVSNGSIKKVSAMQVWTPLNN  52
BAU68120.1         MASFTVAKA---PVA-----A------PKAFK--GLSKAT--KPAAKVANVS--KANEMMVWTPQNN  47
BAI47769.1         MASFTVAKA---PVA-----A------PKAFK--GLSKAT--KPAAKVANVS--KASEMMVWTPLNN  47
BAI47767.1         MASFTVAKA---PVA-----A------PKAFK--GLSKAT--KPAAKVANVS--KASEMMVWTPLNN  47
D0FY41             MASFTVAKA---PVA-----A------PKAFK--GLSKAT--KPAAKVANVS--KASEMMVWTPLNN  47
XP_013900278.1     MALSM-----------------------------K--SA--VARVQAKAPVA--KANKMMVWQPVNN  32
AGH30308.1         MAAAMKSA----VVC-------------RPMA--K--LA--ASSKQAKAPVA--KANKMMVWRPDNN  42
XP_002955679.1     MAAIVAKSSVATAVVRPARSS------VRPVA--VLKPAIKAAP---VASPA--QANKMMVWTPVNN  54
D8UAG9             MAAMVMKSSVATAVVRPARSS------VRPCA--VLKPAVKAAT---VTAPA--QANKMMVWTPVNN  54
XP_001702409.1     MAAVIAKSSVSAAVARPARSS------VRPMA--ALKPAVKAAP---VAAPA--QANQMMVWTPVNN  54
KXZ54261.1         MAAMIAKSSVSAAVARPARSS------ARVSA--VLKPAVKAAP---VAAPS--SANKMMVWTPVNN  54
AAS48503.1         MAALLAKSASVAPMAKASRTA------TKVQA--SLKPAVRA-AP-KAQPAV--RANQMMVWNPINN  55
Q5VBK1             MAALLAKSASVAPMAKASRTA------TKVQA--SLKPAVRA-AP-KAQPAV--RANQMMVWNPINN  55
AAU93597.1         MAALIAKTCP-AVATPISRGS------TKVQA--SLKPAVTRPAPKAQAPAV--RTNQMMVWRPVNN  56
Q5XR40             MAALIAKTCP-AVATPISRGS------TKVQA--SLKPAVTRPAPKAQAPAV--RTNQMMVWRPVNN  56
XP_002500762.1     MAAISA----VAPVL-------------------------NKAPVVSTGKAA--NTNSMMVWQPHGN  36
XP_002501187.1     MAAISA----VAPVL-------------------------NKAPVVSTGKAA--NTNSMMVWQPHGN  36
C1E330             MAAISA----VAPVL-------------------------NKAPVVSTGKAA--NTNSMMVWQPHGN  36
C1N9I4             MAALCS----VAPVV-------------------------AKVPAVSTGKAS--KSSAMQVWNPTNN  36
BAM68686.1         MAAQACI-CAAAPVVSIAKAT---PAKVARPA--ILAPAVNQWAKKTVSNGI--KTSAMMVWTPTNN  59
K7ZLF2             MAAQACI-CAAAPVVSIAKAT---PAKVARPA--ILAPAVNQWAKKTVSNGI--KTSAMMVWTPTNN  59
ACF16408.1         MAAACAS-LLSAPVAVVASAAPA----RSLKA--LAPARAATFAQKTVSNGI--KTRQMMVWQPTNN  58
XP_005648578.1     MAALTAS-LVSCPVAVAAKPSKAGFSGLARVA--LPAKAVPTFAQRTVSNGC--RTRQMLVWEPVDN  62
WP_051016923.1     MAAFTAS-MVSLPAAVATKPSKAGFSGLARVA--LPSKAVPTFAQRTVSNGC--RTRQMLVWEPTDN  62
```

(Land plants)

```
M0THX7             ---------------------MSAAFLSAGAVAGYVGLRADASAKLF-PEKDCSIGWRSRTVSNGFRTR----------CMKTWNPFNN-    57
M7YK88             -MAPA------------------VMASSATTVAPFQGLKSTPGPPANPPPRRPA---SAASATAGGSDACRVLSLRGRTNTKVWPIEGIK    68
J9QE65             -MAPS------------------VMASSATAVAPFQGLKSTASLPVARRS-T-T---SLAKVSNGGRIR----------CMQVWPAYGN-    55
C5Y519             -MAPT------------------VMASSATAVAPFQGLKSTATLPVARRS-T-T---SLAKVSNGGRIR----------CMQVWPAYGN-    55
P05348             -MAPT-----------------VMMASSATAVAPFQGLKSTASLPVARRS-S-R---SLGNVSNGGRIR----------CMQVWPAYGN-    56
J3NCR4             -MAPT------------------VMASSATSVAPFQGLKSTAGMPVSRRSGS-S---SFGNVSNGGRIK----------CMQVWPIEGI-    56
ABR26034.1         -MAPS------------------VMASSATTVAPFQGLKSTAGMPVARRSGN-S---SFGNVSNGGRIR----------CMQVWPIEGI-    56
I1R5H6             -MAPS------------------VMASSATTVAPFQGLKSTAGMPIARRSGN-S---SFGNVSNGGRIR----------CMQVWPIEGI-    56
I1IIT7             -MAPT------------------VMASSATSVAPFQGLKSTAGLPVASRRSS-N---SLGSVSNGGRIR----------CMQVWPIEGI-    56
M8CFA7             -MAPA------------------VMASSATTVAPFQGLKSTAGLPVSRRSGS-A---GLSSVSNGGRIR----------CMQVWPIEGI-    56
Q9ZRS6             -MAPA------------------VMASSATTVAPFQGLKSTAGLPVGRRSGS-A---GLSSVSNGGRIR----------CMQVWPIEGI-    56
F2CUB8             -MAPA------------------VMASSATTVAPFQGLKSTAGLPVSRRS-S-A---SLGRVSNGGRIR----------CMQVWPIEGI-    55
ALS03825.1         -MAPA------------------VMASSATTVAPFQGLKSTAGLPVGRRS-S-G---SLSSVSNGGRIR----------CMQVWPIEGI-    55
M7YY28             -MAPA------------------VMASSASTVAPFQGLKSTAGLPVSRRS-S-G---SLGSVSNGGRIR----------CMQVWPIEGI-    55
D8T7L5             --------MVTGAASSAVIPMAALSTPVAPKVPGFTGLKATTALTAN----KGGLQWSQKTVANGSRVS----------CMLTWTPYNN-    67
Q43110             -MA--TM--LAAMA----VSAPSCAAAAESLSKSFSGLKANTLFAPKCA--------NPLAISNGSRIS----------AMLVWKPYDN-    62
XP_001773332.1     -MASAVV---SVSIVAAAS--PAAVCRESSSVKQFSGLKSTTLFASKARR--------LSSVHNGSRVQ----------CMQVWNPIGQ-    65
A9T3W5             -MASAVV---SVSIVAAAS--PAAVCRESSSVKQFSGLKSTTLFASKARR--------LSSVHNGSRVQ----------CMQVWNPIGQ-    65
W1P273             -MASTVM--VSSAAVASSAAITRTA--PSQYFAPFSGLKSTAAFPVT-KKPTTD---FSSLPSNGGRVQ----------CMKVWEPYNN-    70
P19309             -MASSMM--AS-----TAAAVARAGPAQSSMV-PFNACRSSVPFPAT-RKANNN---LSTLPGNGGRVS----------CMQVWPPEGL-    66
AEJ33935.1         -MASSMM--VS------SAAVARATPAQSNMVAPFNGLRSSAALPAT-RKANAD---LSTLLSNGGKAR----------CMQVWPPEGL-    66
Q106Z5             -MVSSMM--VS-----SAATFTRASPAQSSMVAPFTGLKSASAFPVT-RKPNAD---LSHLPSNGGRVQ----------CMKVWPIEGV-    67
O24634             MAASSTM--LS-----SVATAACAAPAQASMVAPFVGLKSTSAFPVT-QKPATG---LSTLPSNGGRVQ----------CMKVWPIVGL-    68
P16031             -MASSIMALSSTAAVAA-V-AAPSKTGNSNVVSAFTGLKSMAQFPSSKTMSNAGAEWEQKTTSNGSRVR----------CMQVWPPYAN-    76
Q5ZF97             -MA-SSM--LSTSST------AVAGRAQATMVAPYAGLKSTSVFPATRKT---G---NAKLARNAGKVR----------CMQVWPPLGK-    63
V4KM78             -MA-SSV--VSSAATFS-G--VNRIPTQANMVAPFTGLKSSAAFPVT-HKTNFD---ITSLASNGSKVH----------CMQVWPPVGK-    68
M4EYY5             -MA-SVM--LSSA-----T--MASSPAHATMVAPFIGLKSSAAFPVT-CKANTK---VTSITSNGGRVN----------CMKVWPPVGK-    64
NP_974098.1        -MA-SSM--LSSA-----T--MVASPAQATMVAPFNGLKSSAAFPAT-RKANND---ITSITSNGGRVN----------CMQVWPPIGK-    64
V4KN70             -MA-SSM--LSSA-----T--MVASPAQATMVAPFTGLKSSAAFPVT-RKANND---ITSIASNGGRVN----------CMQVWPPIGK-    64
P08135             -MA-SSM--LSSA-----A--VVTSQLQATMVAPFTGLKSSAAFPVT-RKTNTD---ITSIASNGGRVS----------CMKVWPPIGK-    64
D7MJY9             -MA-SSM--LSSA-----A--VVTSPAQATMVAPFTGLKSSATFPVT-RKANND---ITSISSNGGRVS----------CMKVWPPIGK-    64
R0GKZ4             -MA-SSM--LSSA-----A--VVTSPAQATMVAPFTGLKSSAAFPVT-RKANND---ITSIASNGGKVS----------CMKVWPPIGK-    64
Q306K0             -MA-YSM--LSSA-----A--VVTSPAQATMVAPFTGLKSSAAFPVT-RKANND---ITSIASNGGRVS----------CMKVWPPVGK-    64
F5A555             -MA-SSM--LSSA-----A--VVTSPAQATMVAPFTGLKSSAAFPVT-RKANND---ITSIASNGGRVS----------CMKVWPPVGK-    64
Q8VX71             -MA-SSI--MSSATV------ARPSAAQATVIAPFTGLKNIATFPTTRKS---S---DLSFPSNGGKVR----------CMQVWPPEGK-    63
P08705             ----MAS--IS-SSVAT-V--SRTAPAQANMVAPFTGLKSNAAFPTT-KKA-ND---FSTLPSNGGRVQ----------CMKVWPPLGL-    64
Q40250             ----MAS--ISSSAIAT-V--NRTTSTQASLAAPFTGLKSNVAFPVT-KKANND---FSSLPSNGGRVQ----------CMKVWPPIGL-    66
ALJ30131.1         ----MAS--ISSSAVAT-V--NRTTAAQASMVAPFTGLKSSAAFPVT-KKA-ND---FSSLPSNGGRVQ----------CMKVWPPINM-    65
Q84Y16             ----MAS--ISSSAVAT-V--NRTTSAQASMVAPFTGLKSNVAFPVT-KKS-ND---FSSLPSNGGRVQ----------CMKVWPPLGL-    65
P17673             ----MAL--ISSAAVTT-I--NRAP-VQANLATPFTGLKSSAGFPVT-KK-NND---ITSITSNGSRVN----------CMQVWPPVGK-    64
O65194             ----MAL--ISSAAVTT-V--NRASSAQANLVAPFTGLKSSAGFPVT-KKTNND---ITSIASNGGRVN----------CMQVWPPVGK-    66
G7KMR3             ----MAL--ISSAAVTT-V--NRV---SANLVAPFTGLKSSAGFPVT-KKTNND---ITSITSNGGRVN----------CMQVWPPIGK-    63
O65349             -MA-SSV--MSTATVAT-----GANAAQASMIASFNGLKSAASFPVTRK-QDLD---ITSIASNGGRVE----------CMLVWPPINK-    66
NP_001295872.1     -MA-SSI--VSSAAAAT-----RSNVAQASMVAPFTGLKSAASFPVTKKNNNVD---ITSLASNGGRVR----------CMQVWPPINM-    67
P07179             -MA-SSV--ISSAAVAT-----RSNVTQASMVAPFTGLKSSATFPVTKK-QNLD---ITSIASNGGRVS----------CMQVWPPINM-    66
AAA34116.1         -MA-SSV--LSSAAVAT-----RSNVAQANMVAPFTGLKSAASFPVSRK-QNLD---ITSIASNGGRVQ----------CMQVWPPINK-    66
```

P69250	-MA-SSV--LSSAAVAT-----RSNVAQANMVAPFTGLKSAASFPVSRK-QNLD---ITSIASNGGRVQ----------CMQVWPPINK-	66
V9IN82	-MA-SSV--LSSAAVAT-----RSNVAQANMVAPFTGLKSAASFPVSRK-QNLD---ITSIASNGGRVQ----------CMQVWPPINK-	66
P26577	-MA-SSV--MSSAAVAT-----RGNGAQASMVAPFTGLKSTASFPVSRK-QNLD---ITSIASNGGRVR----------CMQVWPPINM-	66
Q41351	-MA-SSI--MSSAAVAT-----RSNGAQASMVAPFTGLKSNASFPVSRK-TNLD---ITSIASNGGRVR----------CMQVWPPINM-	66
Q08184	-MA-SSL--MSNAATTM-A--AATTTAQANMVAPFNGLKSISAFPVT--RKNND---ITSVASNGGRVQ----------CMQVWPPLGM-	67
Q9XGX5	-MA-SSM--MSNAATAV-AVAATSGGAQANMVAPFNGLKSIASFPVT--RKSND---ITSIASNGGRVQ----------CMQVWPPVGK-	69
P07689	---MASM--ISSSAVTT-V--SRASTVQSAAVAPFGGLKSMTGFPVK--KVNTD---ITSITSNGGRVK----------CMQVWPPIGK-	66
NP_001241137.1	-MA-SSM--ISSPAVTT-V--N-RAG--AGMVAPFTGLKSMAGFPTR--KTNND---ITSIASNGGRVQ----------CMQVWPPVGK-	64
Q42815	-MA-SSM--ISSPAVTT-V--N-RAG--AGMVAPFTGLKSMAGFPTR--KTNND---ITSIASNGGRVQ----------CMQVWPPVGK-	64
Q42822	-MA-SSM--ISSPAVTT-V--N-RAG--AGTVAPFTGLKSMAGFPTR--KTNND---IASIASNGGRVQ----------CMQVWPTTGK-	64
Q42823	-MA-SSM--ISSPAVTT-V--N-RAG--AGTVAPFTGLKSMAGFPTR--KTNND---IASIASNGGRVQ----------CMQVWPTTGK-	64
Q9XQB5	-MA-SSM--ISSPAVTT-V--N-RAGA-AGMVAPFTGLKSLGGFPTR--KTNND---ITSVANNGGRVQ----------CMQVWPTIGK-	65
T2DPC1	-MA-SSM--ISSPAVTT-V--N-RAGAGAGMVAPFTGLKSLGGFPSR--KMNND---ITSVANNGGRVQ----------CIQVWPTVGK-	66
M5XB14	-MA-SSM--ISSATVAS-VYADRAAPAQASLVAPFTGLKSASAFPGA--KKTND---ITSIASNGGRVQ----------CMKVWPTVGL-	69
Q02980	-MA-SSM--ISSGTVAT-VSADRPAPAQARMVAPFTGLKSSSASPVT--RKSND---ITSIASNGGRVQ----------CMQVWPPLGL-	69
P24007	-MA-SSM--ISSGTVAT-VSADRPAPAQARMVAPFNGLKSSSAFPVT--RKSND---ITSIASNGGRVQ----------CMQVWPPLGL-	69
Q8GRJ7	-MA-SSM--ISSAAVAT-T--TRASPAQASMVAPFNGLKAASSFPIS--KKSVD---ITSLATNGGRVQ----------CMQVWPPRGL-	67
F8K9Q2	-MA-SSM--ISSAAVAT-T--TRASPAQASMVAPFTGLKAASSFPIS--KKSVD---ITSLATNGGRVQ----------CMQVWPPTGK-	67
Q43832	-MA-SSV--LSSAAVAT-V--S-RTPAQASMVAPFTGLKSTVGFPAT--KKNDD---ITSLASNGGRVQ----------CMKVWPTQNM-	66
D6BR54	-MA-SSM--LSTATVAS-I--NRASPAQATMVAPFTGLKSTAGFPA--RKTTND---ITSIAGNGGRVQ----------CMKVWPTQGL-	67
Q42915	-MA-SSM--LSTATVAS-I--NRVSPAQATMVAPFTGLKSTPVFPTT-RKTNSD---ITSITSNGGKVQ----------CMKVWPTLGM-	68
P29684	-MA-SSM--LSTAAVAC-I--NRASPAQASMVAPFTGLKSTSAFPTT-RKTTTD---ITSIASNGGRVQ----------CMQVWPPRGK-	68
E7E1K9	-MA-SSM--ISSAT--T-V--ARTTGAQASMVAPFTGLKSMATFPATTKKTNND---ITSLASNGGRVQ----------CMQVWPPVGK-	67
D1MZ08	-MASASM--LSS----P-A--VRATPAQATMVAPFTGLKSASAFPVT-RKSSED---ITSLSSNGGRVQ----------CMQVWPPIGK-	65
XP_004135094.1	-MA-SSI--LSSAAVAS-V--NSASPAQASMVAPFTGLKSSAGFPIT-RKNNVD---ITTLASNGGKVQ----------CMKVWPPLGL-	68
P08474	-MA-SSI--LSSAAVAS-V--NSASPAQASMVAPFTGLKSSAGFPIT-RKNNVD---ITTLASNAGKVQ----------CMKVWPPLGL-	68
A9PHQ1	-MA-SSV--ISSAAVAT-V--N-RTPAQANMVAPFNGLKSTSAFPVT-RKANND---ITSIASNGGRVQ----------CMQVWPPTGL-	67
K4HMH5	-MA-SSM--ISSATIA------TASPAQANMVAPFTGLKSASAFPVT-RKANND---ITSLASNGGRVQ----------CMQVWPPTGK-	65
K4HMG9	-MA-SSM--ISSATIAT-V--NCSSPAQANMVAPFTGLKSASAFPVT-RKANND---ITSLASNGGRVQ----------CMQVWPPLGK-	68
B9S5G3	-MA-SSM--ISSAS----V--SRSSPAQATMVAPFTGLKSAASFPVT-RKANND---ITSIASNGGRVQ----------CMQVWPPLGK-	65
A5C718	-MA-SSM--VSSATVAT-I--NRATPAQANMVAPFTGLKSLSTFPGT-RKANTD---ITSVASNGGRVR----------CMKVWPTEGL-	68
Q96542	-MA-CSM--ISSATVAA-V--SRASPAQSSMVAPFTCLKSTSAFPVT-QKTNND---ITSIASNGGRVQ----------CMQVWPPLGL-	68
V4UWB0	-MA-SSM--ISSATVAT-A--NRASLAQASMVAPFTGLKSSSAFPAT-KKTNND---ITSIASNGGRVQ----------CMKVWPPTGL-	68
Q3LFQ4	-MA-SSV--ISSATVAA-V--SRAVPAQASMVAPFTGLKSTAAFPVT-RKV-ND---ITSLPSNGGRVQ----------CMKVWPPLGL-	67
O22077	-MA-SSM--ISSATVAT-V--SRATPAQATMVAPFTGLKSTAAFPAT-RKSNND---ITSLASNGGRVQ----------CMKVWPPLGL-	68
Q64FQ1	-MA-SSM--ISSATVAT-V--SRATPAQATMVAPFTGLKSTAAFPAT-QKSNND---ITSLASNGGRVQ----------CMKVWPPLGL-	68

Figure S2. Phylogenetic analysis of RbcS TPs from algae and higher plants.

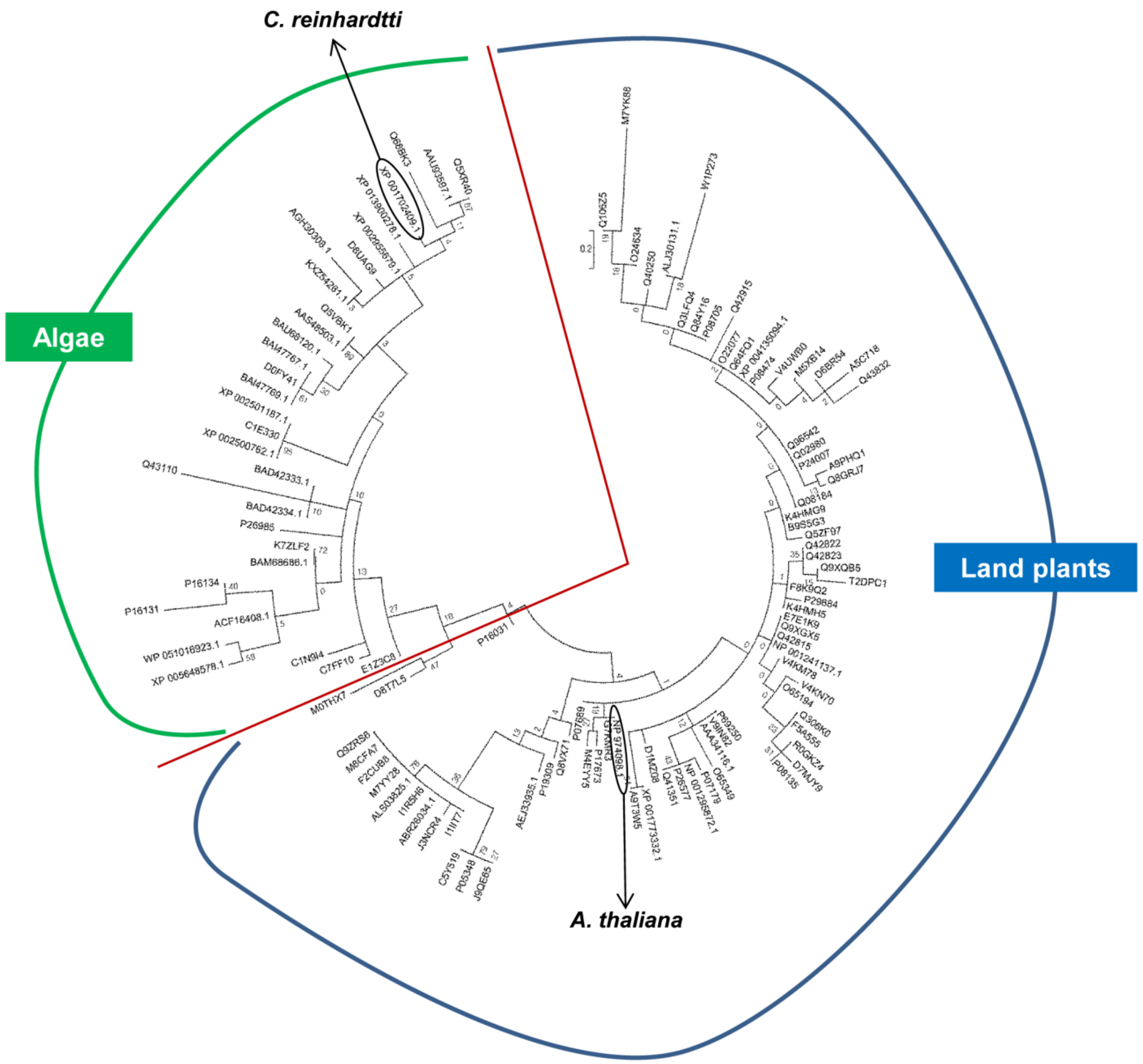

Figure S3. Original gel scan.

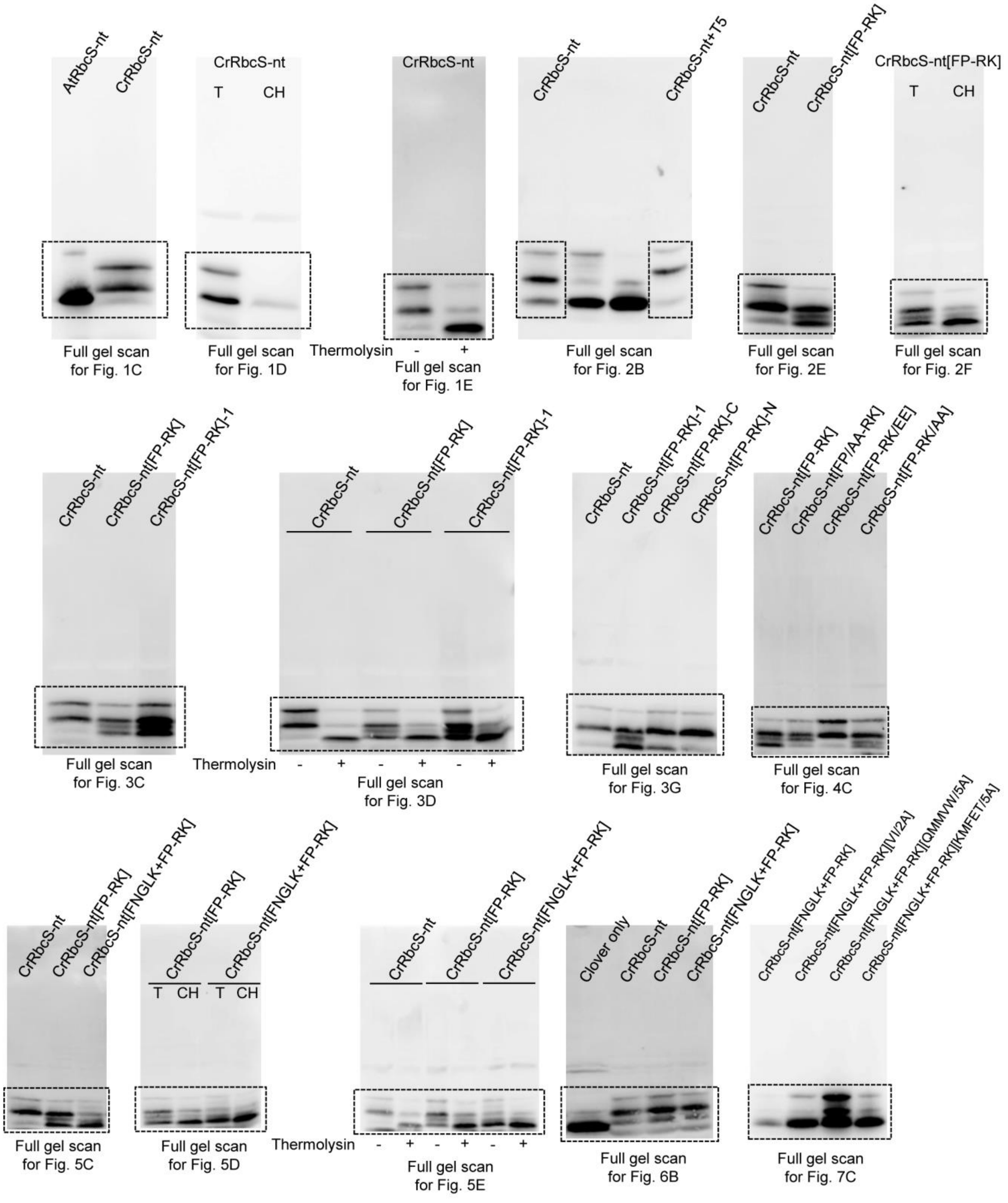

Supplemental reference

1. Tamura, K. et al. MEGA5: molecular evolutionary genetics analysis using maximum likelihood, evolutionary distance, and maximum parsimony methods. Mol Biol Evol. 28(10), 2731-9 (2011).

Table S1. The information for species used for multiple sequence alignments of RbcS TPs

	Accession #	Species
**Algae**	>XP_001702409.1	*Chlamydomonas reinhardtii*
	>XP_002955679.1	*Volvox carteri f. nagariensis*
	>KXZ54261.1	*Gonium pectoral*
	>D8UAG9	*Volvox carteri*
	>AAS48503.1	*Dunaliella tertiolecta*
	>Q5VBK1	*Dunaliella tertiolecta*
	>AAU93597.1	*Dunaliella salina*
	>Q5XR40	*Dunaliella salina*
	>BAU68120.1	*Ulva compressa*
	>BAI47769.1	*Ulva pertusa*
	>BAI47767.1	*Ulva pertusa*
	>D0FY41	*Ulva pertusa*
	>XP_002500762.1	*Micromonas commode*
	>XP_002501187.1	*Micromonas commode*
	>C1E330	*Micromonas sp* (strain RCC299/NOUM17)
	>C1N9I4	*Micromonas pusilla*
	>XP_013900278.1	*Monoraphidium neglectum*
	>AGH30308.1	*Chromochloris zofingiensis*
	>XP_005648578.1	*Coccomyxa subellipsoidea*
	>WP_051016923.1	*Nocardia cyriacigeorgica*
	>BAM68686.1	*Botryococcus braunii*
	>K7ZLF2	*Botryococcus braunii*
	>ACF16408.1	*Lobosphaera incisa*
	>BAD42333.1	*Nannochloris bacillaris*
	>BAD42334.1	*Nannochloris bacillaris*
	>Q68BK3	*Nannochloris bacillaris*
	>C7FF10	*Chlorella pyrenoidosa*
	>E1Z3C8	*Chlorella variabilis*
	>P26985	*Batophora oerstedii*
	>P16131	*Acetabularia peniculus*
	>P16134	*Acetabularia acetabulum*
	>K8F34	*Bathycoccus prasinos*
	>A4SA23	*Ostreococcus lucimarinus*
**Land plants**	>I1IIT7	*Brachypodium distachyon*
	>XP_001773332.1	*Physcomitrella patens*
	>A9T3W5	*Physcomitrella patens*
	>D8T7L5	*Selaginella moellendorffii*
	>P16031	*Larix laricina*
	>Q43110	*Pteris vittata*
	>M0THX7	*Musa acuminata subsp. malaccensis*
	>NP_974098.1	*Arabidopsis thaliana*
	>Q306K0	*Brassica napus*
	>R0GKZ4	*Capsella rubella*
	>F5A555	*Brassica juncea*
	>D7MJY9	*Arabidopsis lyrata*
	>V4KN70	*Thellungiella salsuginea*
	>P08135	*Raphanus sativus*
	>K4HMH5	*Gossypium raimondii*
	>K4HMG9	*Gossypium darwinii*
	>B9S5G3	*Ricinus communis*
	>O22077	*Fagus crenata* (Japanese beech)
	>Q64FQ1	*Fagus sylvatica* (Beechnut)
	>V4UWB0	*Corchorus olitorius*
	>Q96542	*Betula pendula*
	>A9PHQ1	*Populus trichocarpa*
	>A5C718	*Vitis vinifera* (Grape)
	>Q3LFQ4	*Panax ginseng*
	>Q42915	*Manihot esculenta* (Cassava)
	>P29684	*Hevea brasiliensis* (Para rubber tree)
	>D6BR54	*Jatropha curcas* (Barbados nut)
	>Q43832	*Spinacia oleracea* (Spinach)
	>XP_004135094.1	*Cucumis sativus*
	>P08474	*Cucumis sativus* (Cucumber)

	>M5XB14	*Prunus persica*
	>Q02980	*Malus sp. (Crab apple)*
	>P24007	*Pyrus pyrifolia* (Chinese pear)
	>Q8GRJ7	*Coffea arabica* (Arabian coffee)
	>F8K9Q2	*Coffea canephora* (Robusta coffee)
	>NP_001295872.1	*Solanum lycopersicum*
	>AAA34116.1	*Nicotiana tabacum*
	>P69250	*Nicotiana sylvestris*
	>V9IN82	*Nicotiana attenuata*
	>AAA33719.1	Petunia
	>P07179	*Solanum lycopersicum*
	>P26577	*Solanum tuberosum*
	>Q41351	*Stellaria longipes* (Longstalk starwort)
	>O65349	*Capsicum annuum* (Bell pepper)
	>D1MZ08	*Eucalyptus globulus*
	>Q106Z5	*Musa acuminata*
	>NP_001241137.1	*Glycme max*
	>Q42815	*Glycine soja*
	>Q42822	*Glycine tomentella* (Woolly glycine)
	>Q42823	*Glycine tabacina*
	>Q9XQB5	*Vigna radiata var. radiata* (Mung bean)
	>T2DPC1	*Phaseolus vulgaris* (Kidney bean)
	>P17673	*Trifolium repens* (Creeping white clover)
	>O65194	*Medicago sativa*
	>G7KMR3	*Medicago truncatula*
	>E7E1K9	*Litchi chinensis* (Lychee)
	>ALJ30131.1	*Tragopogon dubius*
	>Q84Y16	*Chrysanthemum morifolium* (Florist's daisy)
	>P08705	*Helianthus annuus*
	>Q40250	*Lactuca sativa* (Garden lettuce)
	>Q08184	*Mesembryanthemum crystallinum*
	>Q9XGX5	*Amaranthus hypochondriacus*
	>M4EYY5	*Brassica rapa subsp. pekinensis*
	>V4KM78	*Thellungiella salsugineaL*
	>P07689	*Pisum sativum* (Garden pea)
	>W1P273	*Amborella trichopoda*
	>Q8VX71	*Rumex obtusifolius* (Bitter dock)
	>O24634	*Fritillaria agrestis*
	>Q5ZF97	*Plantago major* (Common plantain)
	>AEJ33935.1	*Wolffia Australiana*
	>P19309	*Lemna gibba* (Swollen duckweed)
	>ABR26034.1	*Oryza sativa*
	>I1R5H6	*Oryza glaberrima*
	>J3NCR4	*Oryza brachyantha*
	>ALS03825.1	*Triticum aestivum*
	>M7YK88	*Triticum urartu*
	>M7YY28	*Triticum urartu*
	>F2CUB8	*Hordeum vulgare var. distichum*
	>M8CFA7	*Aegilops tauschii*
	>Q9ZRS6	*Secale cereale*
	>J9QE65	*Saccharum hybrid cultivar GT28*
	>C5Y519	*Sorghum bicolor*
	>P05348	*Zea mays*

Table S2. Primer sequences used to generate the constructs in this study

CaMV 35S-T	TTTCAGAAAGAATGCTAACC
nosT-B	GAACGATCGGGGAAATTC
Primer1	GCTCAAGCAAACCAAATGATGGTATGGACACCAGTAAACAACAAGATGTTCGAAACTTTCAGTTATCTG CCTCCTCTTACTGATCTCGAGATCCAAGGAGAT
primer2	AGCTAGAAGTTCAGTAAGACCAATGGCTGCACTAAAGCCAGCAGTAAAGGCTGCTCCAGTAGC TGCACCAGCTCAAGCAAACCAAATGAT
Primer3	GCTCTAGAATGGCTGCAGTTATTGCTAAATCATCAGTATCAGCTGCTGTAGCTAGACCAG
CrRbcS_FNGLK-F	AGAAGTTCAGTAAGACCA TTCAACGGA CTAAAGCCAGCAGTAAAG
CrRbcS_FNGLK-R	CTTTACTGCTGGCTTTAGTCCGTTGAATGGTCTTACTGAACTTCT
CrRbcS-atRbcS-T5-F	GACATTACTTCCATCACAAGCAACGGCGGA AACCAAATGATGGTATGG
CrRbcS-atRbcS-T5-R	TCCGCCGTTGCTTGTGATGGAAGTAATGTC TGCTTGAGCTGGTGCAGC
CrRbcS_FP-RK-F	AAGCCAGCAGTAAAGGCTTTCCCAGTAGCTCGCAAGGCTCAAGCAAACCAAATG-F
CrRbcS_FP-RK-R	CATTTGGTTTGCTTGAGCCTTGCGAGCTACTGGGAAAGCCTTTACTGCTGGCTT-R
CrRbcS_FP-RK`1-F	AGACCAATGGCTGCACTATTCCCAGCCCGCAAGGCTGCTCCAGTAGCTGCA
CrRbcS_FP-RK`1-R	TGCAGCTACTGGAGCAGCCTTGCGGGCTGGGAATAGTGCAGCCATTGGTCT
CrRbcS_FP-RK-C-F	AACCAAATGATGGTATGGTTCCCAGTAAACCGCAAGATGTTCGAAACTTTCAGT
CrRbcS_FP-RK-C-R	ACTGAAAGTTTCGAACATCTTGCGGTTTACTGGGAACCATACCATCATTTGGTT
CrRbcS_FP-RK-N-F	GCTAAATCATCAGTATCATTCCCAGTAGCTCGCAAGGCTAGAAGTTCAGTAAGA
CrRbcS_FP-RK-N-R	TCTTACTGAACTTCTAGCCTTGCGAGCTACTGGGAATGATACTGATGATTTAGC
CrRbcS FP/AA-RK-F	AAGCCAGCAGTAAAGGCT GCTGCT GTAGCTCGCAAGGCTCAA
CrRbcS FP/AA-RK-R	TTGAGCCTTGCGAGCTACAGCAGCAGCCTTTACTGCTGGCTT
CrRbcS FP-RK/AA-F	AAGGCTTTCCCAGTAGCT GCTGCT GCTCAAGCAAACCAAATG
CrRbcS FP-RK/AA-R	CATTTGGTTTGCTTGAGCAGCAGCAGCTACTGGGAAAGCCTT
CrRbcS FP-RK/EE-F	AAGGCTTTCCCAGTAGCT GAAGAAGCTCAAGCAAACCAAATG
CrRbcS FP-RK/EE-R	CATTTGGTTTGCTTGAGCTTCTTCAGCTACTGGGAAAGCCTT
CrRbcS (VI/2A)-F	GC TCTAGA ATGGCTGCA GCTGCA GCTAAATCATCAGTATCA
CrRbcS (QMMVW/5A)-F	CGCAAGGCTCAAGCAAAC GCTGCA GCTGCA GCA ACACCAGTAAACAACAAG
CrRbcS (QMMVW/5A)-R	CTTGTTGTTTACTGGTGTTGCTGCAGCTGCAGCGTTTGCTTGAGCCTTGCG
CrRbcS (KMFET/5A)-F	TGGACACCAGTAAACAAC GCTGCA GCTGCA GCA TTCAGTTATCTGCCTCCT
CrRbcS (KMFET/5A)-R	AGGAGGCAGATAACTGAATGCTGCAGCTGCAGCGTTGTTTACTGGTGTCCA
